# Regular versus Irregular Exercise Differentially Modulates Hippocampal‐Hepatic Acetylcholine Flux to Coordinate Fear Memory Extinction and Liver Inflammation

**DOI:** 10.1002/advs.202500177

**Published:** 2025-09-26

**Authors:** Pengjiao Ma, Kuan Zhang, Long Yi, Chuanyan Yang, Hedong Lang, Jianbin Sun, Jin Li, Qian Bai, Yuhang He, Shuang‐Shuang Dai, Man‐Tian Mi

**Affiliations:** ^1^ Department of Biochemistry and Molecular Biology School of Basic Medical Science Army Medical University Chongqing 400038 China; ^2^ Research Center for Nutrition and Food Safety Chongqing Key Laboratory of Nutrition and Health Institute of Military Preventive Medicine Army Medical University Chongqing 400038 China; ^3^ Brain Research Center and State Key Laboratory of Trauma and Chemical Poisoning Army Medical University Chongqing 400038 China; ^4^ Department of Neurosurgery Southwest Hospital Army Medical University Chongqing 400038 China

**Keywords:** acetylcholine, fear of memory extinction, hepatic inflammation, irregular exercise, regular exercise

## Abstract

Despite the known benefits of exercise, an in‐depth comparison of the effects of regular versus irregular exercise on brain–body interactions and molecular mechanisms remains lacking. This study demonstrates that regular exercise robustly enhances fear memory extinction in mice, whereas irregular exercise has only mild effects. This divergence arises from regular rather than irregular exercise strongly inhibiting axonal mRNA transport and local translation of choline acetyltransferase (ChAT) in septal cholinergic neurons projecting to the hippocampus, thereby reducing hippocampal acetylcholine (ACh) levels and inhibiting α7 nicotinic acetylcholine receptor (α7‐nAChR) activation on astrocytes. Critically, this hippocampal ACh‐α7‐nAChR signaling modulates hepatic ACh via the amygdala–dorsal motor nucleus of the vagus (DMV)–hepatic vagus circuit. Irregular exercise inhibits this pathway inadequately, so that increasing hepatic ACh flux and recruiting a novel neutrophil subset characterized by high expression of F‐box and leucine‐rich repeat protein 6 (FBXL6^high^). Subsequent abnormal iron transport from these neutrophils to hepatocytes promotes the metabolism of arachidonic acid into the proinflammatory mediators prostaglandin H2 (PGH2) and leukotriene B4 (LTB4), exacerbating nonalcoholic fatty liver disease. The findings elucidate the essential mechanisms underlying the neurometabolic benefits of regular exercise and the pathological risks of irregular exercise, offering transformative insights for preventive strategies.

## Graphical abstract

Ma *et al.* report the hippocampal and hepatic acetylcholine (ACh) levels are differently modulated by regular versus irregular exercise, which contributes to coordinate hippocampal astrocyte activity and hepatic FBXL6^high^ neutrophil recruitment. This ACh‐modulated brain‐liver circuit links the effects of exercise on fear memory extinction and liver inflammation.

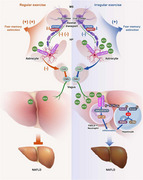

## 1 Introduction

Although it is widely acknowledged that exercise provides numerous benefits to the human body, the effects of exercise on the body in relation to the balance between training variables—volume, intensity, and frequency—are complex and multifaceted and involve physiological, neurological, and psychological adaptations.^[^ ^1^, ^2^, ^3^, ^4^ ^]^ Understanding how these variables interact is crucial for optimizing performance, preventing injury, and promoting long‐term health. In contemporary society, factors such as evolving fast‐paced lifestyles and societal demands make it challenging to maintain consistent engagement in structured physical activity. Compared with regular exercise (EX‐R), a typical exercise program involving fixed‐intensity exercise on set days, irregular exercise (EX‐IR), characterized by intermittent periods of activity interspersed within fragmented schedules, is becoming increasingly prevalent because of its alignment with the demands of hectic modern routines.^[^ ^5^ ^]^ However, to accomplish the intended training volume over a specified period, little is known about whether EX‐R or EX‐IR is beneficial or detrimental. To address this issue, this study was conducted to explore murine models of EX‐R and EX‐IR in the form of treadmill running. A better understanding of these biological processes and the underlying mechanisms would better serve public health by strengthening the recommendations to manage a balanced daily life with exercise and even provide a foundation for the development of exercise‐mimetic pharmacological interventions.

Exercise perturbs multiple systems from the whole‐body level to the molecular level in an integrated manner. The central motor drive and central command can be “fine‐tuned” via feedback signals that monitor substrate levels, mean arterial pressure (MAP), blood gases, pH, fluid status, and body temperature despite the marked homeostasis challenges associated with exercise.^[^ ^5^ ^]^ In addition, increasing evidence suggests that the biological mechanisms underlying the effects of exercise and distinct exercise modalities on organisms involve brain–body interactions via multidimensional regulation of neural–metabolic–immune axes.^[^ ^6^, ^7^, ^8^ ^]^ However, in‐depth fundamental knowledge of the molecular and cellular mechanisms through which exercise‐sensitive neural circuits orchestrate brain–body interactions and functions remains largely lacking. To address these issues, here, we focused on comparing the effects of EX‐R and EX‐IR on cognitive function, including fear responses, spatial learning and memory, anxiety, spatial fear memory extinction, metabolism of major organs, such as the liver and heart, and immune status, particularly immune cell heterogeneity in circulation and its impact on nonalcoholic fatty liver disease (NAFLD). The molecules and pathways associated with EX‐R and EX‐IR were systematically investigated, and the results are expected to provide valuable insights into the molecular mechanisms underlying the regulation of homeostasis through different exercise modes and identify potential limitations. Understanding these biological processes and the underlying mechanisms would better serve public health by strengthening the recommendations to manage a balanced daily life with exercise and even provide a foundation for the development of exercise‐mimetic pharmacological interventions.

## 2 Results

### 2.1 Exercise Efficiently Improves Fear Memory Extinction

**Figure 1:**
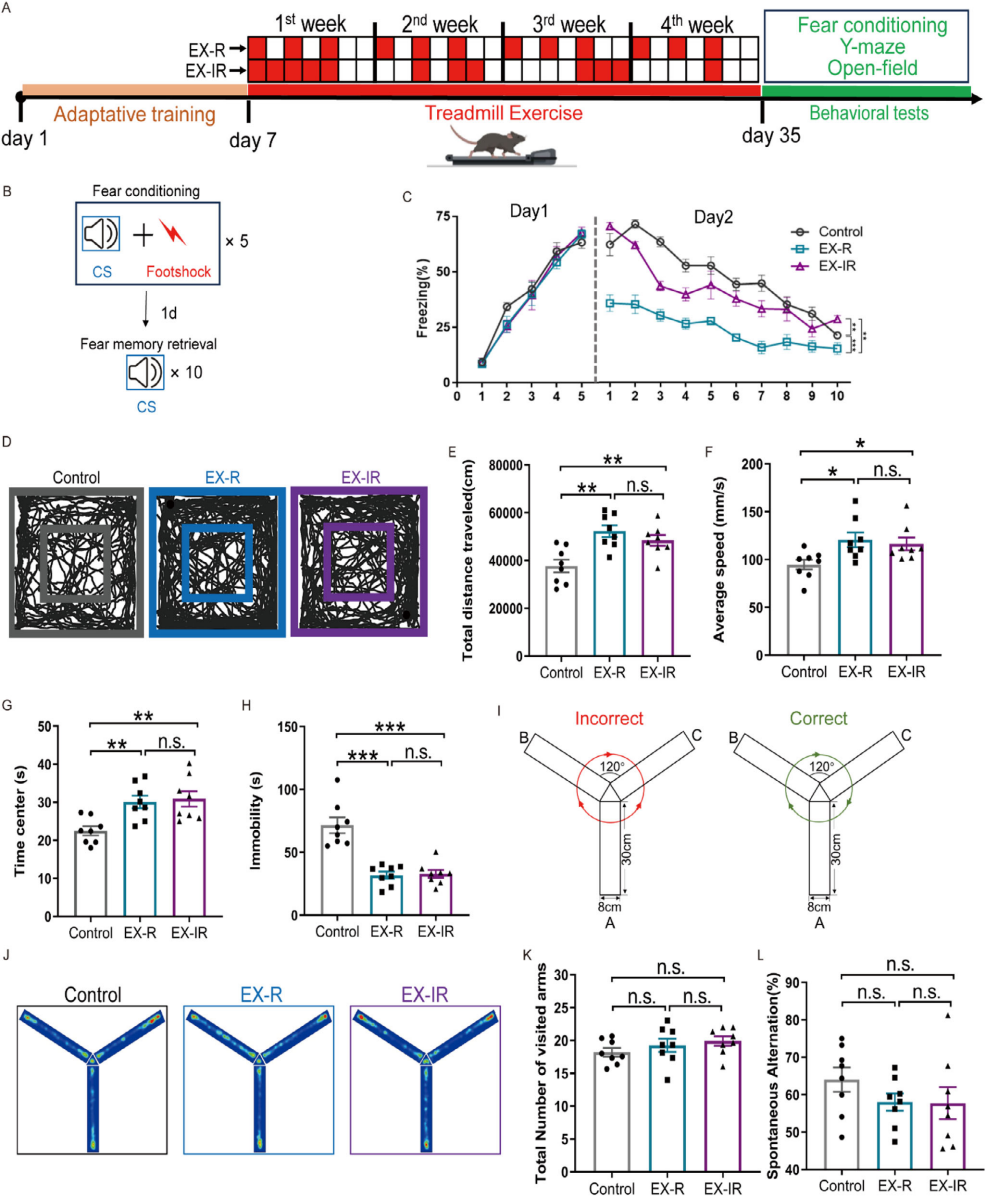
Effects of regular versus irregular exercise on the cognitive behaviors of mice. A) Protocols for treadmill exercise and behavioral tests in C57BL/6 mice. B) Fear conditioning protocol. C) Freezing levels during conditioning and retrieval (*n* = 6 mice in each group). D) Traces of mice in the open field test. E) Summary of total distance travelled in the open field test. *n* = 8 mice in each group. F) Summary of the average speed in the open field test. *n* = 8 mice in each group. G) Summary of time spent in the center of the open field test. *n* = 8 mice in each group. H) Summary of immobility time in the open field test. *n* = 8 mice in each group. I) Diagram of the Y‐maze test. J) Trajectory heatmap of mice in the Y‐maze. Each picture represents the path result for one mouse. K) Statistics on the total number of visited arms. *n* = 8 mice in each group. L) Statistics on spontaneous alternation. *n* = 8 mice in each group. The data are presented as the mean ± SEM. SEM, standard error of the mean. *P < 0.05, **P < 0.01, ***P < 0.001. n.s., no significant difference.

The effects of EX‐R and EX‐IR on the behaviors of mice, specifically fear memory, spatial memory, and anxiety, were preliminarily explored (**Figure** **1**A). We conducted a series of assessments, including fear memory evaluation, Y‐maze assessment, and open field tests. During the fear memory evaluations, the mice were subjected to protocols for both fear conditioning and retrieval (Figure 1B). The fear memory extinction test was conducted on day 2 of the experimental procedure, in accordance with prior studies.^[^ ^9^, ^10^ ^]^ The data indicate that EX‐R not only enhances the extinction of fear memory but also inhibits the retrieval of fear memory compared with those in the EX‐IR and control groups (mice without any exercise) (Figure 1C). In addition, both the EX‐R and EX‐IR groups demonstrated significant increases in activity levels in the open field, as evidenced by the total distance travelled, average speed and time center, which were notably greater than those of the control groups (Figure 1D–G), whereas immobility was significantly reduced in these groups (Figure 1H). These findings are corroborated by reports indicating that mice exhibit increased exercise capacity following four weeks of moderate‐intensity treadmill exercise.^[^ ^11^ ^]^ Furthermore, anxiety levels were diminished in both the EX‐R and EX‐IR groups, which aligns with previous studies that have shown reduced anxiety in mice after exercise.^[^ ^12^, ^13^ ^]^ In the Y‐maze experiment, which evaluated spatial memory, no significant differences were detected among the three groups (Figure 1I–L). These results suggest that while both regular and irregular exercise can inhibit the persistence of fear memories, regular exercise is much more effective at facilitating the extinction of such memories.

### 2.2 Reduction of Hippocampal ACh Level Diminishing ACh‐α7‐nAChR Signaling in Astrocytes Contributes to Exercise‐Mediated Improvement in Fear Memory Extinction

**Figure 2:**
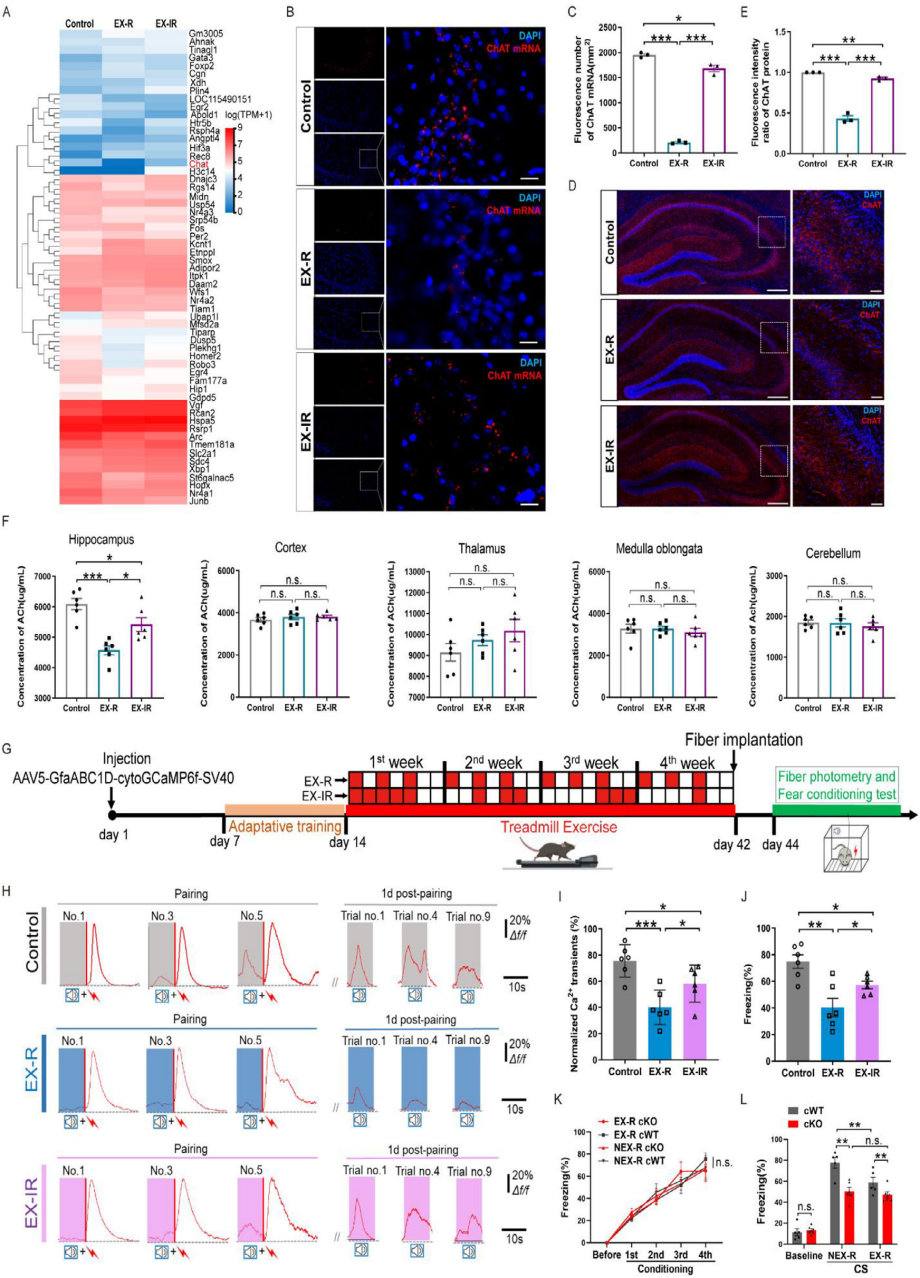
Associations between fear memory extinction and changes in ChAT mRNA expression, ACh levels and ACh‐α7‐nAChR signaling in astrocytes in the hippocampus induced by regular exercise versus irregular exercise. A) Heatmap of DEGs in the hippocampal area in the three different groups (*n* = 3 mice in each group). Red denotes upregulated DEGs, and blue denotes downregulated DEGs. B) The expression of ChAT mRNA (red) in the hippocampus of mice was visualized by in situ hybridization using RNAscope technology. Nuclei were stained with DAPI (blue). Scale bar, 50 µm. C) Fluorescence intensity statistics of ChAT mRNA in the hippocampus region. D) Immunofluorescence of the hippocampal region from mice (Control/EX‐R/EX‐IR groups) was performed, and the ChAT protein (magenta) was immunostained and counterstained with DAPI (blue). Scale bar, 250 µm; zoom in to 50 µm. E) Fluorescence intensity data for ChAT protein expression in the hippocampus. F) The level of ACh in different brain regions was detected by ELISA. G) Protocols for the fear conditioning tests. H) Astrocytic Ca^2+^ transients in response to CS^+^ during pairing and 1d postpairing. I) Bar graphs summarizing the amplitudes of astrocytic Ca^2+^ transients in response to CS^+^ during pairing and 1 d postpairing. J) Summary of the freezing levels in response to the CS^+^ at 1 d postpairing. *n* = 6 mice in each group. K) Freezing levels of cWT and α7‐nAChR cKO (cKO) mice during pairing. *n* = 6 mice in each group. L) Freezing levels of cWT and α7‐nAChR cKO (cKO) mice at 1 d postretrieval. *n* = 6 mice in each group. The data are presented as the mean ± SEM. SEM, standard error of the mean. *P < 0.05, **P < 0.01, ***P < 0.001. n.s., no significant difference.

To investigate the mechanism of fear memory extinction induced by regular exercise, we performed transcriptome sequencing of various brain regions, including the cortex (I and II), hippocampus (III), and thalamus (IV) (Figure S1A, Supporting Information). By integrating transcriptomics with metabolomics, we performed a comprehensive analysis of the changes in the transcriptional activity of genes related to alterations in metabolite levels. A heatmap of the transcriptomic DEGs in the hippocampus indicated that acetylcholinetransferase (ChAT) mRNA levels in the hippocampus region of the regular and irregular exercise groups were significantly lower than those in the control group (**Figure** **2**A). Notably, no such changes in ChAT mRNA were observed in other brain regions (Figure S1B–G, Supporting Information). Further validation through RNAscope experiments confirmed the decreased levels of ChAT mRNA in the hippocampal region of mice subjected to both regular and irregular exercise (Figure 2B,C). Consistent with the observed changes in ChAT mRNA levels, decreases in ChAT protein levels in the hippocampus were detected by fluorescence and western blot in the exercise groups (Figure 2D,E; Figure S1F, Supporting Information). Given that ChAT is the primary enzyme responsible for the synthesis of acetylcholine (ACh), we subsequently assessed ACh levels in brain tissues. There was also a decrease in ACh in the murine hippocampus in both the regular and irregular exercise groups, while no significant differences were observed in other brain regions (Figure 2F). Furthermore, we aimed to evaluate changes in ACh synthesis and its transport and metabolic pathways (high‐affinity choline transporter, CHT1 and acetylcholinesterase, AChE) and did not observe any significant differences (Figure S1G–J, Supporting Information). These findings suggest that exercise specifically induced a reduction in ChAT, which contributes to the downregulation of ACh levels in the hippocampus. Notably, both the mRNA and protein expression of ChAT and the ACh level decreased significantly more in the regular exercise group than in the irregular exercise group (Figure 2B–F). These findings align with the observation that regular exercise has a markedly more efficient effect than irregular exercise on promoting fear memory extinction in mice. It has been well established that ACh in the brain is involved in the regulation of attention, memory formation, stress response, and pain processing in rodents.^[^ ^14^ ^]^ One study reported that during treadmill running, global changes in ACh are subject to local adjustments, leading to spatially heterogeneous ACh release.^[^ ^15^ ^]^ However, the factors that may determine the local and global dynamics of ACh concentrations are currently unclear. The essential enzyme ChAT for ACh synthesis is an important marker that determines its distribution. Taken together, these findings suggest that the distribution of ChAT in the hippocampus is the main cause of fear memory fading in our exercise models.

Given that our previous study demonstrated the essential role of α7‐nAChRs on astrocytes in the persistence of fear memory^[^ ^16^ ^]^ and ACh is the natural ligand for α7‐nAChRs, we subsequently investigated whether exercise induced changes in ChAT and ACh levels, which would affect ACh‐α7‐nAChR activity in hippocampal astrocytes, thereby contributing to exercise‐regulated fear memory extinction. We employed an optic fiber photometer, and the diagram is shown in Figure 2G and Figure S2A (Supporting Information). As previously reported,^[^ ^16^ ^]^ this method requires the chronic implantation of a fine optical fiber into the right dorsal hippocampus region. AAV‐mediated, astrocyte‐specific expression of GCaMP6f enabled us to capture Ca^2+^ transients that reflected the activities of astrocytes labelled with GCaMP6f. Immunostaining further validated the specificity of GCaMP6f expression for astrocytes and revealed the precise locations of the recording sites within the dorsal hippocampus region (Figure S2B, Supporting Information).

Representative recordings demonstrated that the conditioned stimulus (CS) effectively induced persistent astrocytic Ca^2+^ transients in the control group during the one‐day post‐training phase, which corresponds to the fear memory retrieval stage. In contrast, astrocytic Ca^2+^ transients significantly decreased during the fear memory retrieval stage in the EX‐R group (Figure 2H, I). Consistent with these findings, the freezing level in the EX‐R group was also lower than that in the control group (Figure 2J). Furthermore, compared with those in the EX‐R group, the amplitudes of astrocytic Ca^2+^ transients and the degree of freezing in the EX‐IR group were not significantly reduced (Figure 2H–J). These results preliminarily confirm that the reduction in hippocampal astrocyte activation is involved in promoting fear memory extinction following EX‐R. Furthermore, mice with a conditional knockout (KO) of α7‐nAChR in hippocampal astrocytes exhibited effects similar to those of exercise on fear memory extinction. (Figure 2K,L; Figure S2C–D, Supporting Information). Taken together, these results suggest that decreased ACh levels in the hippocampus reduce the activation of α7‐nAChR on hippocampal astrocytes, which mediates the improvement in fear memory extinction following exercise training.

### 2.3 Inhibiting the Axonal Transport and Local Translation of ChAT mRNA in Cholinergic Neurons in the Medial Septum That Project to the Hippocampus Mediates the Exercise‐Induced Reduction in Hippocampal ACh Levels

**Figure 3:**
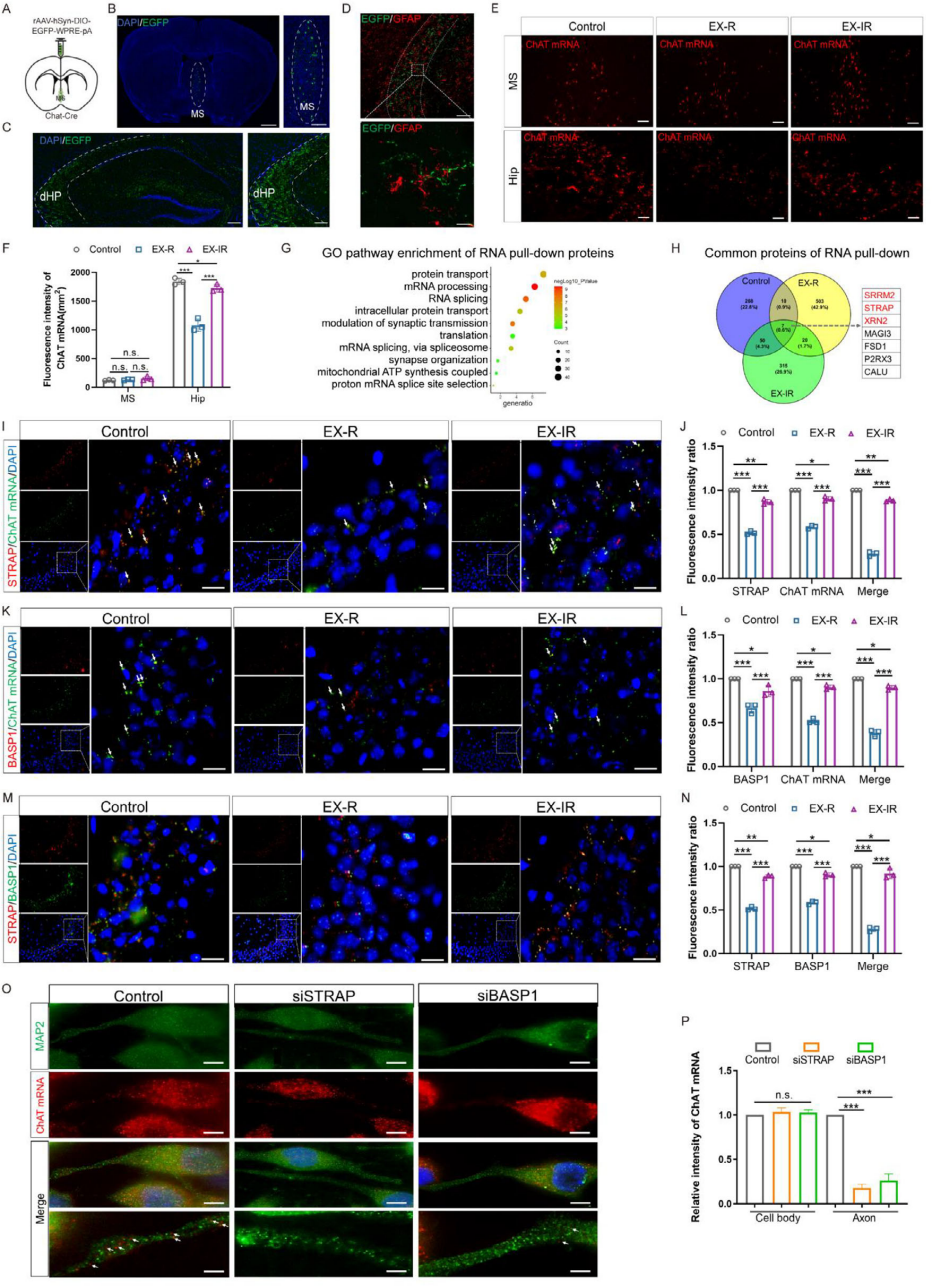
Exercise‐induced changes in and mechanisms of ChAT mRNA axonal transport in the cholinergic neurons of the medial septum projecting to the hippocampus. A) rAAV‐hSyn‐DIO‐EGFP‐WPRE‐pA injection scheme for the MS of CHAT‐Cre mice. B) Composite confocal image showing the selective targeting of MS cholinergic neurons in ChAT‐Cre mice. MS, medial septum; LSI, lateral septum intermediate; VDB, ventral diagonal band. Scale bar, 1 mm (coronal) and 100 µm (zoomed in). C) Composite confocal image of prominent MS cholinergic projections to the hippocampus. Scale bar, 250 µm; zoom in to 100 µm. D) Confocal images of astrocytes labelled with rAAV‐hSyn‐DIO‐EGFP‐WPRE‐pA (green) in the hippocampus. Scale bars, 20 µm (top) and 5 µm (bottom). E) Expression of ChAT mRNAs (red) in regions of the MS and dHP, as determined using RNAscope technology; scale bar, 100 µm. F) Fluorescence intensity statistics of ChAT mRNAs (red) in the MS and dHP, *n* = 3. G) GO pathway enrichment of RNA pull‐down proteins, i.e., proteins that interact with ChAT mRNA in the brain. H) Venn diagram for common proteins identified by RNA pull‐down. The proteins common to the three groups involved in RNA modification function are shown in red, and those not involved are shown in black. I) Immunostaining of STRAP and ChAT mRNA in the hippocampus; scale bar, 10 µm. J) Fluorescence colocalization statistics of STRAP and ChAT mRNA in the hippocampus. K) Immunostaining of BASPI and ChAT mRNA in the hippocampus; scale bar, 10 µm. L) Fluorescence colocalization statistics of BASPI and ChAT mRNA in the hippocampus. M) Immunostaining of STRAP and BASPI in the hippocampus; scale bar, 10 µm. N) Fluorescence colocalization statistics of STRAP and BASPI in the hippocampus. O) ChAT mRNA in axons and growth cones. NG108‐15 cells were transfected with a siRNA construct directed against STRAP/BASP1 (siSTRAP/siBASP1) or a control (untransfected), and 24 h after transfection, the cells were fixed and hybridized with probes specific for ChAT mRNA (red) and MAP2 (green) using RNAscope technology. Scale bar, 5 µm. P) Fluorescence intensity was quantified in the cell body and axons (*n* = 10 cells from three independent experiments). The data are presented as the mean ± SEM. SEM, standard error of the mean. *P < 0.05, **P < 0.01, ***P < 0.001. n.s., no significant difference.

Considering that the exercise‐induced decrease in ACh contributes to the enhancement of fear memory extinction through the modulation of ACh‐α7‐nAChR on hippocampal astrocytes, we subsequently explored the underlying mechanisms of the reduction in ACh induced by exercise in greater depth. Previous studies have demonstrated that the medial septum (MS) is a major source of cholinergic innervation to the hippocampus and that cholinergic neurons located in the MS predominantly project their axons to the hippocampus, forming septo‐hippocampus pathways for the release of ACh in the hippocampus. A recent study further revealed that cholinergic input into the hippocampus from the MS drives astrocytes to regulate fear extinction by activating α4/α7‐nAChR.^[^ ^10^ ^]^ Our study aimed to determine whether EX‐R and EX‐IR directly reduce the activation effect of α7‐nAChR by altering the distribution of ChAT mRNA in MS‐hippocampus projections, accelerating fear memory extinction. To analyze the transport of ChAT mRNA along axons during the projection of MS neurons to the hippocampus, we delineated the projection patterns of MS cholinergic neurons into the dentate gyrus (dHP). To selectively label cholinergic neurons, we introduced a Cre‐dependent AAV expressing EGFP (rAAV‐hSyn‐DIO‐EGFP‐WPRE‐pA) into the MS of chat‐Cre mice (**Figure** **3**A,B). We subsequently examined the projections in the dHP (Figure 3C) and confirmed that the axons of cholinergic neurons projecting from the MS closely surrounded the cell bodies and processes of astrocytes in the hippocampus (Figure 3D). Simultaneously, we utilized RNAscope to evaluate the levels of ChAT mRNA in both the MS and dHP regions. Our results indicated that the fluorescence of ChAT mRNA in the MS remained stable across all three experimental groups. In contrast, a notable decrease was observed in the dHP region of the EX‐R group, whereas a mild reduction was noted in the EX‐IR group (Figure 3E,F). These findings suggest that the concentration of ChAT mRNA in the neuronal cell bodies of MS patients remained unchanged following exercise. However, compared with that in the irregular exercise group, the transport of this mRNA to the hippocampus via axonal pathways was significantly diminished, particularly in the regular exercise group.

To investigate the underlying mechanisms of ChAT mRNA axonal transport, we performed RNA pull‐down followed by sequencing to identify binding proteins associated with ChAT mRNA. KEGG pathway enrichment analysis revealed that the mRNA processing pathway was the most significantly represented pathway, and seven proteins were consistently detected across all three experimental groups (Figure 3G,H). The three proteins included RNA‐binding protein serine/threonine kinase receptor‐associated protein (STRAP), serine/arginine repetitive matrix protein 2 (SRRM2), and 5'‐3' exoribonuclease 2 (XRN2) (**Table** **1**). Therefore, we focused on the three RNA‐binding proteins. Immunofluorescence assays revealed that STRAP colocalized with ChAT mRNA and was markedly reduced in the EX‐R group but slightly decreased in the EX‐IR group, whereas the expressions of the other two RNA‐binding proteins did not significantly differ (Figure 3I,J; Figure S3A,B, Supporting Information), indicating that STRAP plays a role in the axonal localization of ChAT mRNA. Additionally, mass spectrometry, molecular docking, and Co‐IP analyses revealed that brain acid‐soluble protein 1 (BASP1), a neurotrophic factor closely associated with axonal growth and plasticity, potentially interacts with STRAP1 (Figure S3C–E, Supporting Information). Next, we confirmed that BASP1 colocalized with both ChAT mRNA and STRAP, with a notable decrease in the EX‐R group (Figure 3K,L). In addition, the colocalization of STRAP and BASP1 was significantly weaker in the EX‐R group (Figure 3M,N). This finding was further supported by the results of in vitro siRNA experiments (Figure 3O,P) and the effects of BASP1 cKO on neurons in vivo (Figure S3F,G, Supporting Information). These findings suggest that the STRAP‐BASP1 complex contributes to the axonal transport of ChAT mRNA such that the deficiency of the complex after exercise, especially regular exercise, suppresses the axonal transport of ChAT mRNA from the MS to the hippocampus.

**Table 1:** Information of the seven common proteins among the Control, EX‐R, EX‐IR groups identified by RNA pull‐down assay (as indicated in Figure 3H).

Protein	Full name	Uniport ID
MAGI3	Membrane‐associated guanylate kinase, WW and PDZ domain‐containing protein 3;	Q9EQJ9
FSD1	Fibronectin type III and SPRY domain‐containing protein 1	Q7TPM6
P2RX3	P2X purinoceptor 3	Q3UR32
CALU	Calumenin	O35887
SRRM2	Serine/arginine repetitive matrix protein 2	Q8BTI8
STRAP	Serine‐threonine kinase receptor‐associated protein	Q9Z1Z2
XRN2	5'‐3' exoribonuclease 2	Q9DBR1

### 2.4 The Regular Exercise‐Diminished Activation of α7‐nAChR in Hippocampal Astrocytes Also Suppresses Hepatic ACh Release via the Hippocampal Astrocyte/Amygdala/Hepatic Vagus Nerve Terminal Circuit to Control the Hepatic Inflammatory Response after Exercise

**Figure 4:**
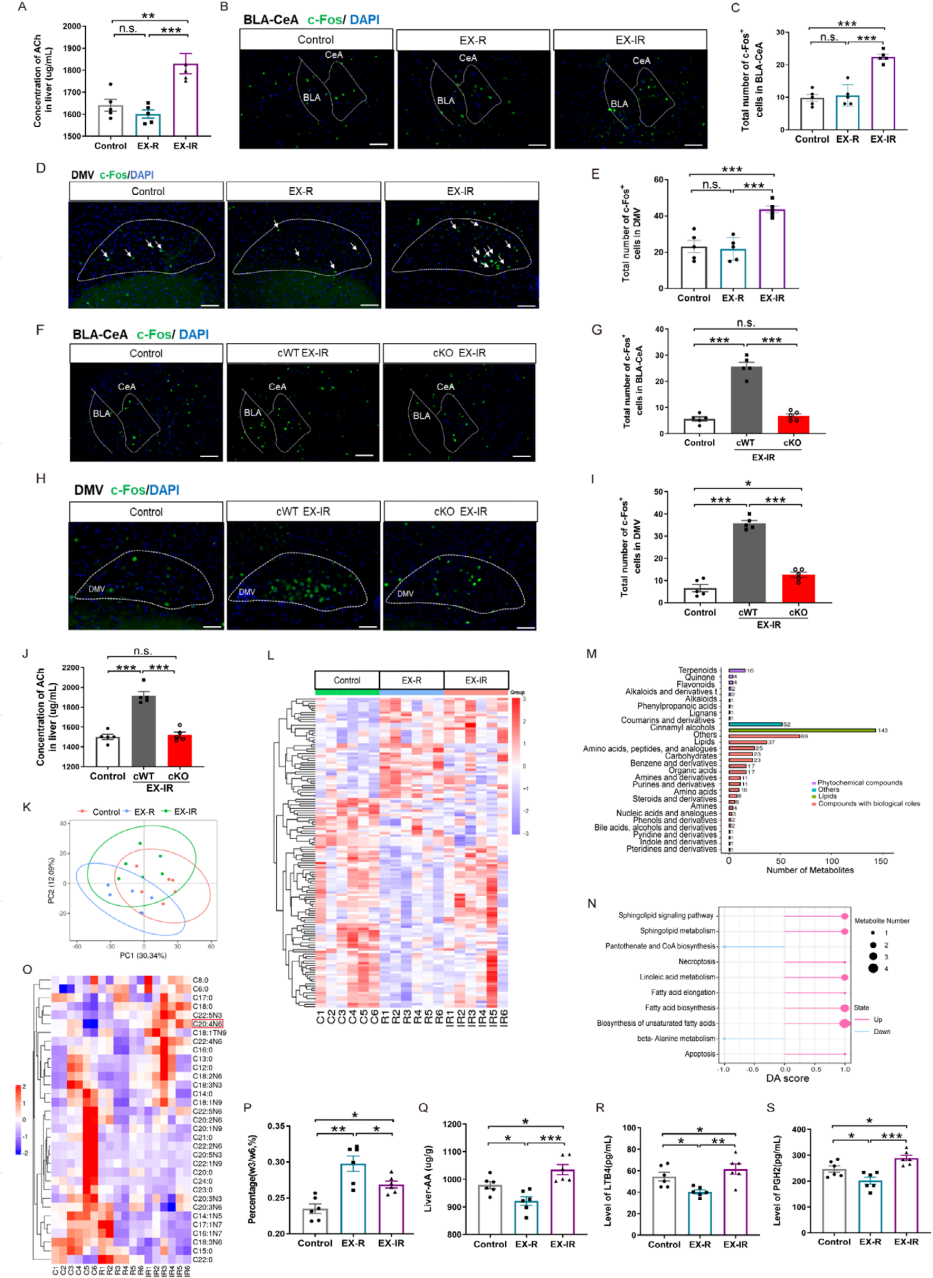
Linkage of exercise‐modulated ACh‐α7‐nAChR signaling in hippocampal astrocytes and hepatic ACh release and its associated hepatic long‐chain fatty acid metabolism. A) Hepatic ACh levels were detected by ELISA; *n* = 5 in each group. B) Fluorescence of c‐Fos^+^ in the CeA. Scale bar, 100 µm. C) Fluorescence statistics of c‐Fos^+^ cells in the CeA; *n* = 5 in each group. D) c‐Fos^+^ fluorescence in the DMV. The arrows represent c‐Fos^+^ neurons; scale bar, 100 µm. E) Fluorescence statistics of c‐Fos^+^ cells in the DMV. *n* = 5 in each group. F) c‐Fos^+^ fluorescence in the CeA of α7‐nAChR cKO (cKO) mice after EX‐IR. Scale bar, 100 µm. G) Fluorescence images of c‐Fos^+^ cells in the CeA of α7‐nAChR cKO (cKO) mice after EX‐IR. *n* = 5 in each group. H) c‐Fos^+^ fluorescence in the DMV of α7‐nAChR cKO (cKO) mice after EX‐IR. Scale bar, 100 µm. I) Fluorescence images of c‐Fos^+^ cells in the DMV of α7‐nAChR cKO mice (cKO) after EX‐IR. *n* = 5 in each group. J) Hepatic ACh levels in α7‐nAChR cKO mice (cKO) were detected by ELISA after EX‐IR treatment. *n* = 5 in each group. K) Liver metabolomics sample distribution. Partial least squares discriminant analysis representation of replicates from the control (red dots), EX‐R (blue dots), and EX‐IR (green dots) utricles using the 500 most variable metabolites in the dataset. Six mice for each sample were collected. L) Heatmap displaying the relative levels of liver metabolites in the control, EX‐R and EX‐IR groups. Significant metabolites with positive and negative effects are depicted in red and purple, respectively. M) The number of metabolites whose abundance significantly increased or decreased is plotted according to the differential types (see color code). Each group had six repeated samples. N) Comparison of the differential abundance (DA) scores of metabolic pathways between EX‐IR and EX‐R samples on the basis of metabolite abundance. The DA score captures the average gross changes for all metabolites in a pathway. A score of 1 indicates that all measured metabolites in the pathway increase in EX‐IR compared with EX‐R, and a score of −1 indicates that all measured metabolites in a pathway decrease. Pathways with no fewer than three measured metabolites were used for the DA score calculation. O) Heatmap of long‐chain fatty acids targeting metabolomic differences in the liver. The samples were from the control, EX‐R and EX‐IR groups, and each group had six repeated samples. P) Ratio of long‐chain fatty acids w3/w6 in the liver; *n* = 6. Q) The level of arachidonic acid in the liver was detected by ELISA in the control, EX‐R and EX‐IR groups (*n* = 6). R,S) The levels of PGH2 and LTB4 in the liver, including those in the control, EX‐R and EX‐IR groups, were detected by ELISA (*n* = 6). The data are presented as the mean ± SEM. SEM, standard error of the mean. *P < 0.05, **P < 0.01, ***P < 0.001. n.s., no significant difference.

Multiple organ systems are affected by exercise, initiating diverse homeostatic responses. Among them, the liver‒brain axis involves cross‐talk between the liver and the brain through the nervous, circulatory, and endocrine systems and is considered among the important pathways for systematically changing internal stability and sustainably impacting the metabolism and immunity of the body. Considering the significant role of ACh in peripheral organs, we examined whether both regular and irregular modes of exercise induce changes in peripheral ACh levels in the liver. To our surprise, the changes in hepatic ACh in the murine EX‐R and EX‐IR groups were inconsistent. EX‐R did not significantly affect hepatic ACh, whereas EX‐IR markedly elevated hepatic ACh, in contrast to its regulatory effect in the brain (**Figure** **4**A). Considering that hippocampus–amygdala circuits and amygdala–peripheral vagus circuits have been reported in other models of disease^[^ ^17^, ^18^, ^19^, ^20^ ^]^ and that ACh is the main neurotransmitter of the vagus nerve, we investigated whether the hippocampus–amygdala–hepatic vagus circuit is involved in exercise‐induced changes in ACh levels in the liver. The results indicated that compared with that in the control group, c‐Fos expression in the amygdala of murine brain tissues did not significantly change in the EX‐R group but markedly increased in the EX‐IR group (Figure 4B,C). This consistent trend was also observed in the dorsal motor nucleus of the vagus (DMV) nuclei (Figure 4D,E), which is known to be involved in vagal excitatory efferent pathways to peripheral organs.^[^ ^21^ ^]^

In EX‐IR mice with cKO α7‐nAChR on hippocampal astrocytes, EX‐IR‐induced c‐Fos expression in the amygdala and DMV was abolished, and hepatic ACh expression was reduced (Figure 4F–J). In addition, after the nAChR inhibitor methyltobacillus (MLA) was injected into the dorsal hippocampus, the c‐Fos levels in the amygdala and DMV significantly decreased (Figure S4A, Supporting Information). These data indicated that regular exercise rather than irregular exercise diminished the ACh‐α7‐nAChR signal on hippocampal astrocytes and was involved in the inhibition of hepatic ACh through the suppression of the hippocampus–amygdala–hepatic vagus circuit. To our surprise, the metabolomics results revealed that the two exercise modes significantly altered liver metabolites (Figure 4K,L). The most enriched substances were classified as lipids, and the fatty acid synthesis and elongation‐related pathways were highly enriched (Figure 4M,N). Therefore, we further performed fatty acid‐targeted metabolomics analysis and reported that the well‐known beneficial w3/w6 ratio was significantly increased in the liver of EX‐R mice (Figure 4O,P), whereas the levels of arachidonic acid (AA) and its metabolic inflammatory products prostaglandin H2 (PGH2) and leukotriene B4 (LTB4) were increased in the liver of EX‐IR mice; these data demonstrate that EX‐IR induced liver inflammation (Figure 4Q–S). By analyzing multiple previous reports concerning the side effects of exercise on inflammation and the anti‐inflammatory effects,^[^ ^22^, ^23^, ^24^ ^]^ our data support the view that exercise patterns influence the ability of exercise to modulate organ inflammation. Therefore, here, we speculate that exercise (especially irregular exercise) causes more or less inflammation in the liver, but the regularity of exercise is efficient for eliminating potential exercise‐associated side effects, which needs further investigation.

### 2.5 The Recruited Neutrophil in Liver Induced by Irregular Exercise Is A Novel FBXL6^high^ Neutrophil Subset

**Figure 5:**
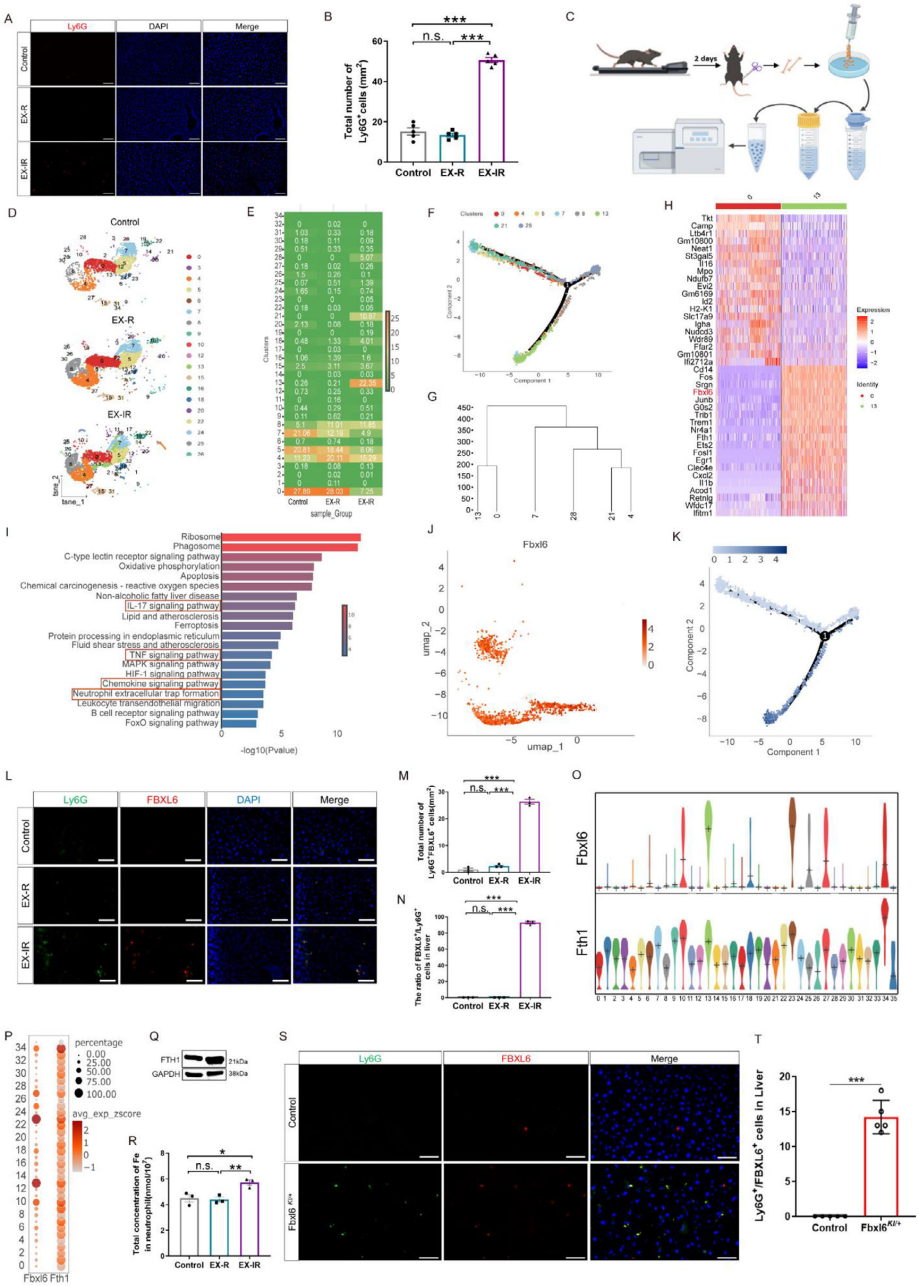
Characteristics of the FBXL6^high^ neutrophil subset induced by irregular exercise in mice. A) Fluorescence of Ly6G (red) and nuclei (DAPI, blue) in the liver. Scale bar, 100 µm. B) Fluorescence statistics of Ly6G^+^ cells in the liver; *n* = 5 in each group. C) Schematic diagram of mouse bone marrow neutrophil extraction. D) The t‐SNE (t‐distributed stochastic neighbor embedding) plot illustrates the clustering of single‐cell transcriptomic data from mouse bone marrow neutrophil samples, which were isolated from the control, EX‐R and EX‐IR groups (*n* = 3 in each group). E) Heatmap of cell cluster proportions. The number is the ratio of clusters to total neutrophils. F) Pseudotime trajectories of differential clusters. Each dot represents a single cell. G) Cluster tree of differential clusters. The dendrogram represents the hierarchical relationship between the clusters, with the height of each fusion point indicating the distance at which the clusters were merged. H) Heatmap of marker genes for the 13 clusters. Rows represent individual genes, and columns represent clusters 0 and 13. Gene expression levels are color coded: red indicates high expression, and blue indicates low expression. I) The enrichment of the top 20 pathways of 13 cluster marker genes. J) UMAPs showing the expression pattern of the FBXL6 gene in cluster 13. Each dot represents a single cell. Cells are colored according to the expression level of FBXL6, with a gradient from light (low expression) to red (high expression). K) Dot plot representing the expression trajectory of the FBXL6 gene in the differential clusters (i.e., the subpopulation shown in panel (F)). L) Immunostaining of Ly6G (green), FBXL6 (red), and nuclei (DAPI, blue) in the liver. Scale bar, 100 µm. M) Fluorescence statistics of Ly6G^+^ FBXL6^+^ cells in the liver; *n* = 3. N) The ratio of FBXL6^+^ cells to Ly6G^+^ cells infiltrating the liver; *n* = 3. O) Violin plots of FBXL6 and Fth1 gene expression in different clusters. P) Bubble plots of FBXL6 and Fth1 gene expression in different clusters. Q) FTH1 expression levels were detected by western blotting. R) Intracellular iron colorimetric assay showing the concentration of total Fe in FBXL6^high^ neutrophils from the bone marrow of irregularly exercised mice. S) Immunostaining of Ly6G (green), FBXL6 (red), and nuclei (DAPI, blue) in livers from Fbxl6*^KI/^* ^+^ and control (C57BL/6) mice. T) Fluorescence statistics of Ly6G^+^FBXL6^+^ cells in the livers of Fbxl6*^KI/^* ^+^ and control (C57BL/6) mice. The data are presented as the mean ± SEM. SEM, standard error of the mean. *P < 0.05, **P < 0.01, ***P < 0.001. n.s., no significant difference.

In addition to elevated hepatic ACh levels, we also observed obvious neutrophil infiltration in the livers of EX‐IR group mice but not EX‐R group mice (**Figure** **5**A,B). Therefore, we further analyzed the characteristics of neutrophils whose infiltration was induced by EX‐IR. Using single‐cell RNA sequencing (scRNA‐seq), we analyzed neutrophil heterogeneity in the bone marrow of the experimental mice (Figure 5C,D). The results revealed that the number of neutrophil clusters in the EX‐IR group significantly differed from that in the other two groups, and a relatively large novel cluster, cluster 13, was specifically present among the neutrophils in the EX‐IR group (Figure 5E). The cell trajectories and proportions suggested that cluster 13 may have transformed from the largest cluster (cluster 1) (Figure 5F,G). The gene profile of cluster 13 included high expression of *Cd14*; Fos proto‐oncogene (*Fos*); serglycin (*Srgn*); F‐box and leucine‐rich repeat 6 (*Fbxl6*); the classic proinflammatory cytokines associated with neutrophils, including *Cd14*, *Fos*, *Cxcl2*, and *Il1b*; and transcription factors, such as *Fos*, *Junb*, *Egr1*, and *Ets2*; and *Ifitm1*, which is associated with cell adhesion (Figure 5H). Consistent with these findings, KEGG pathway enrichment analysis revealed that the most relevant pathways were those involved in inflammatory signaling, ferroptosis and regulation (Figure 5I). In addition, we found that FBXL6 was highly expressed in cluster 13, which was completely consistent with the differentiation trajectory (Figure 5F,J,K), as determined by flow cytometry (Figure S4D, Supporting Information). According to the BioGPS database, FBXL6 is barely expressed in normal neutrophils in either humans or mice (Figure S4B,C, Supporting Information); therefore, we suspected that high expression of FBXL6 could serve as a specific marker for detecting neutrophil cluster 13 induced by EX‐IR, and we confirmed that ≈80% of the hepatic‐infiltrated neutrophils highly expressed FBXL6 (Figure 5L–N). Another signature gene for FBXL6^high^ neutrophils is *Fth1* (Figure 5O,P), encoding the protein ferritin heavy chain (FTH1), which functions in iron storage and has ferroxidase activity.^[^ ^25^ ^]^ Compared with those in normal neutrophils, we detected high levels of intracellular iron ions (Fe) and FTH1 in FBXL6^high^ neutrophils (Figure 5Q,R), suggesting that its function is closely associated with Fe regulation. We established a transgenic mouse model with high neutrophil‐specific expression of FBXL6, named FBXL6^KI/+^, and found that even without exercise, FBXL6‐positive neutrophils significantly infiltrated the liver (Figure 5S,T). Taken together, these data revealed that EX‐IR, rather than EX‐R, induced the infiltration of a unique FBXL6^high^ neutrophil subset rich in intracellular Fe with proinflammatory properties in the livers of mice.

### 2.6 Irregular Exercise‐Induced Hepatic ACh Interacts with the Recruited FBXL6^high^ Neutrophils to Promote Inflammation and Lipid Deposition

**Figure 6:**
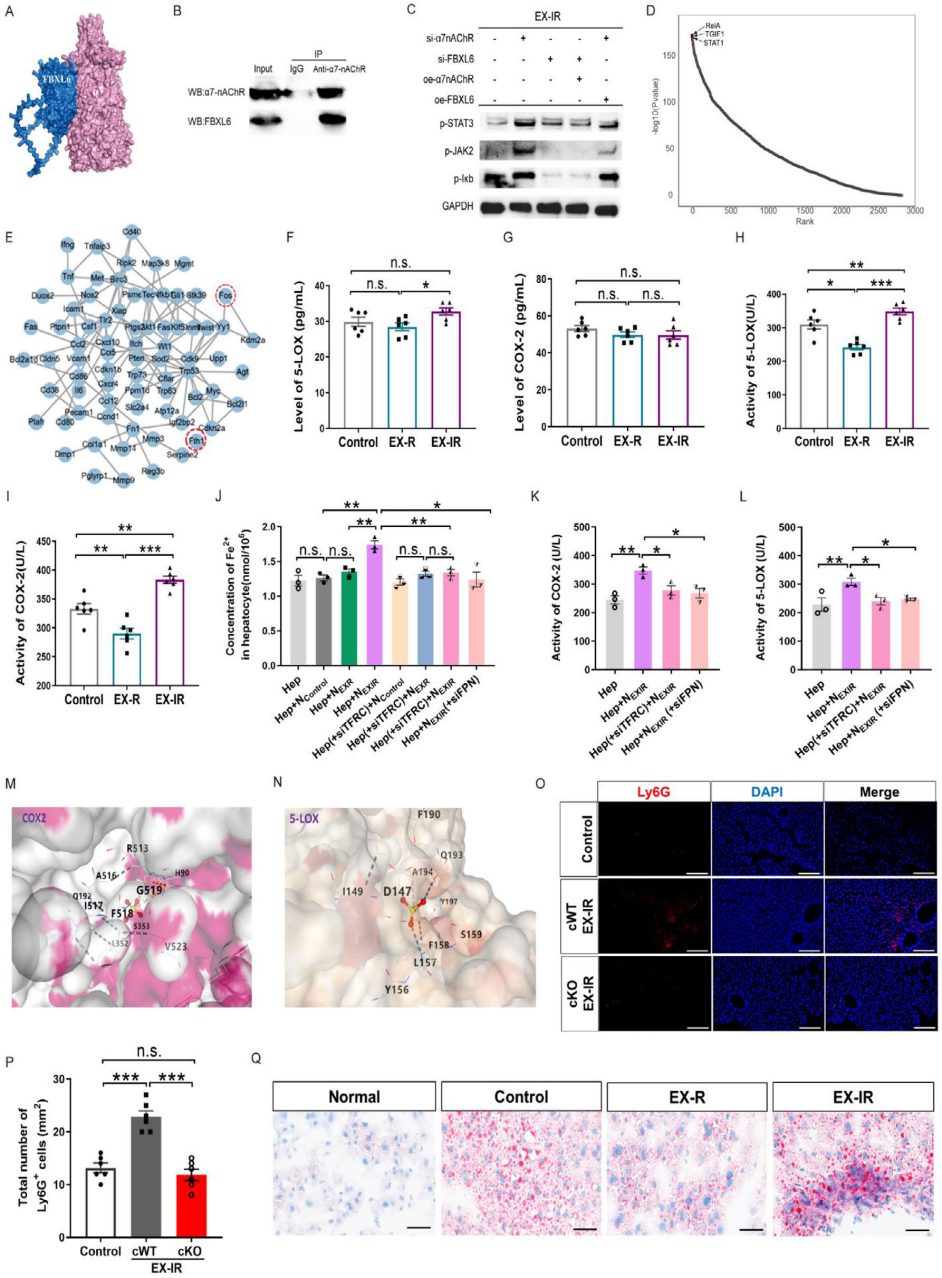
Effects and mechanisms of FBXL6^high^ neutrophils on inflammatory metabolism in hepatocytes and their implications in a murine model of NAFLD. A) Visualization of the interaction between FBXL6 and α7‐nAChR using PyMOL software. B) Co‐IP of FBXL6 and α7‐nAChR in neutrophils from bone marrow after EX‐IR. C) Western blot analysis of the levels of proteins involved in the inflammatory pathway downstream of α7‐nAChR in FBXL6^high^ neutrophils. D) Common transcription factor prediction of the marker genes for FBXL6^high^ neutrophils. E) The TRRUST database was used to predict the NF‐kb target genes. F) The level of 5‐LOX in the liver was detected by ELISA; *n* = 6. G) The level of COX‐2 in the liver was detected by ELISA; *n* = 6. H) The activity of 5‐LOX in the liver was detected by ELISA; *n* = 6. I) The activity of COX‐2 in the liver was detected by ELISA; *n* = 6. J) In vitro coculture of neutrophils and hepatocytes to test iron transport. *n* = 3. K) COX‐2 activity in hepatocytes after coculture of neutrophils and hepatocytes. *n* = 3. L) 5‐LOX activity in hepatocytes after coculture of neutrophils and hepatocytes. *n* = 3. M) CB‐Dock2 analysis of the amino acid interaction sites of Fe^2+^ with COX‐2. N) CB‐Dock2 analysis of the amino acid interaction sites of Fe^2+^ with 5‐LOX. O) Fluorescence of Ly6G (red) and nuclei (DAPI, blue) in the livers of α7‐nAChR cKO mice (cKO) after EX‐IR; scale bar, 100 µm. P) Fluorescence images of Ly6G^+ cells^ in the liver. *n* = 6 in each group. Q) Oil red O staining of the livers of NAFLD mice. Scale bar, 50 µm. The data are presented as the mean ± SEM. SEM, standard error of the mean. *P < 0.05, **P < 0.01, ***P < 0.001. n.s., no significant difference.

Furthermore, we investigated the relationships among the increase in ACh levels, obvious infiltration of FBXL6^high^ neutrophils and increased AA proinflammatory metabolism in the liver induced by EX‐IR. The protein expression database and initial GRAMM docking data revealed a potential interaction between FBXL6 and α7‐nAChRs (**Figure** **6**A), which was confirmed in FBXL6^high^ neutrophils via co‐immunoprecipitation (Co‐IP) experiments (Figure 6B). In addition, we found that FBXL6 interacted with α7‐nAChRs to inhibit Janus kinase 2 (JAK2)–signal transducer and activator of transcription 3 (STAT3) phosphorylation downstream of ACh–α7‐nAChR signaling, which promoted NF‐κB activation in FBXL6^high^ neutrophils (Figure 6C). This interaction, along with the activation of the downstream signaling pathway, was eliminated when the region on the α7‐nAChR responsible for mediating its interaction with FBXL6 was mutated (**Table** **2**; Figure S6A, Supporting Information). Transcription factor analysis revealed that the other signature genes of FBXL6^high^ neutrophils, including *Fth1*, *Cxcl2*, *Il1b*, Cd14, *Fos*, and *Junb*, are target genes of NF‐κB (Figure 6D,E). Since FTH1 functions in iron storage and has ferroxidase activity, which can promote the absorption of surrounding iron, the activation of NF‐κB to increase *Fth1* expression could explain the high levels of Fe in FBXL6^high^ neutrophils. These data indicate that the increase in hepatic ACh levels induced by EX‐IR is responsible for high Fe levels in FBXL6^high^ neutrophils and increased proinflammatory activity in the liver.

**Table 2:** Chrna7 mus truncated sequence.

Name	5′–3′
Chrna7 mus truncated	ATGTGCGGCCGGCGGGGAGGCATCTGGCTGGCTCTGGCCGCGGCGCTGCTGCACGTGTCCCTGCAAGGCGAGTTCCAGAGGAGGCTGTACAAGGAGCTGGTCAAGAACTACAACCCGCTGGAGAGGCCGGTGGCCAACGACTCGCAGCCGCTCACCGTGTACTTCTCCCTGAGCCTCCTGCAGATCATGGATGTGGATGAGAAGAACCAAGTTTTAACCACCAACATTTGGCTACAAATGTCTTGGACAGATCACTATTTGCAGTGGAACATGTCTGAGTACCCCGGAGTGAAAAATGTTCGTTTTCCAGATGGCCAGATTTGGAAACCAGACATTCTCCTCTATAACAGTGCAGATGAACGCTTTGATGCCACATTCCACACCAACGTCTTGGTGAATGCATCTGGGCATTGCCAGTATCTCCCTCCAGGCATATTCAAGAGCTCCTGCTACATCGATGTACGCTGGTTCCCTTTTGATGTGCAGCAGT GCAAACTGAAGTTTGGGTCCTGGTCCTATGGAGGGTGGTCCTTGGACCTGCAGATGCAAGAGGCAGATATCAGCAGCTATATCCCCAATGGAGAATGGGATCTCATGGGAATCCCTGGCAAAAGGAATGAGAAGTTCTATGAATGCTGCAAAGAGCCATACCCAGATGTCACCTACACAGTAACCATGCGCCGTAGGACATAA

Consistent with the findings of the metabolomic analysis, which revealed an increase in the proinflammatory metabolism of AA in the EX‐IR group, we also detected that the activities rather than the expression of cyclooxygenase‐2 (COX‐2) and 5‐lipoxygenase (5‐LOX), key enzymes of AA metabolism, were significantly increased in the liver, whereas the activity of both mediators was decreased in the EX‐R group (Figure 6F–I). COX‐2 and 5‐LOX are usually not activated by inflammatory cytokines, whereas Fe acts as a co‐factor to promote the activation of COX‐2 and 5‐LOX.^[^ ^26^ ^]^ Surprisingly, in the in vitro coculture system of FBXL6^high^ neutrophils and hepatocytes, we found that the Fe released by FBXL6^high^ neutrophils via ferroportin (FPN), a cell surface transmembrane protein and the only known source of nonheme‐based iron export, is accepted by hepatocytes via transferrin receptor protein 1 (TFRC), which imports iron from the extracellular environment into cells; this process contributes to the activation of COX‐2 and 5‐LOX in hepatocytes (Figure 6J–L). We used CB‐Dock2 analysis to confirm the effect of Fe binding to the enzyme active center (Figure 6M,N). Taken together, these data indicate that elevated hepatic ACh levels regulate the Fe metabolism of infiltrated FBXL6^high^ neutrophils to increase proinflammatory AA metabolism in hepatocytes after EX‐IR. More interestingly, the cKO of α7‐nAChRs on hippocampal astrocytes also decreased neutrophil infiltration in the EX‐IR group (Figure 6O,P), which was accompanied by the suppression of the hippocampus–amygdala–DMV–hepatic vagus circuit, which controls the increase in hepatic ACh levels (as shown in Figure 4F–J). Although the underlying mechanism requires further investigation, on the basis of these data, we speculate that because EX‐R does not induce this effect, it may result from controlled c‐Fos expression in the central nucleus of the amygdala (CeA) and the DMV, which are closely associated with no change in hepatic ACh levels and a lack of neutrophil infiltration. These data further demonstrate that exercise‐modulated brain‒liver connections and interactions efficiently orchestrate benefits or harm to the whole body, which is to some extent dependent on the exercise mode, such as regularity or irregularity.

In addition to its role in normal health, exercise is also used as a treatment for liver‐related metabolic diseases. Accordingly, we compared the effects of EX‐R and EX‐IR with those of physically inactive NAFLD mice. Neither EX‐R nor EX‐IR affected basic metabolic indices, inflammatory cytokine expression or lipid deposition (Figure S5A–C, Supporting Information) in healthy mice. However, in NAFLD mice, EX‐R significantly relieved lipid deposition in the liver, which is consistent with the results of several previous studies. In contrast, compared with the control group, EX‐IR aggravated the hepatic accumulation of lipid products (Figure 6Q). However, no FBXL6^high^ neutrophils were found in existing NAFLD databases; thus, these cells should be induced under EX‐IR conditions. To comprehensively assess liver function, we further tested biochemical markers related to liver function in the blood, and the results revealed that alanine aminotransferase (ALT) and aspartate aminotransferase (AST)/ALT levels were significantly higher in EX‐IR mice than in EX‐R mice, indicating more severe hepatic injury (Figure S5D, Supporting Information). We used two complementary experimental approaches: First, we isolated FBXL6^high^ neutrophils from EXIR‐trained mice and intravenously injected them into NAFLD mice, and the results revealed that liver lipid deposition was more severe in NAFLD mice than in control mice. Conversely, we administered FBXL6 siRNA to NAFLD mice prior to EX‐IR training and found that NAFLD‐associated hepatic inflammation, including lipid deposition and the AA/TB4/PGH2 concentration, was significantly reduced (Figure S5E,F, Supporting Information). In addition, we used flow cytometry to sort FBXL6^high^ neutrophils from the EX‐IR mice and co‐culture with the primary hepatocytes from NAFLD mice. We found that the lipid inflammatory mediators in hepatocytes from NAFLD mice were significantly increased after co‐cultured with FBXL6^high^ neutrophils from the EX‐IR mice; however, FBXL6^high^ neutrophils pre‐treated with siRNAs of α7‐nAChR did not elicit this effect on the hepatocytes derived from NAFLD mice (Figure S6B, Supporting Information). These findings not only demonstrate the exacerbating impact of EX‐IR‐induced FBXL6^high^ neutrophil on NAFLD, but also confirmed that the functional interaction between FBXL6 and α7‐nAChRs in FBXL6^high^ neutrophil contributes to its deleterious effect.

In support of our findings, although the optimal frequency and duration of exercise for patients with NAFLD have not been determined, regular exercise training has a long‐term therapeutic effect by reducing fat accumulation and inflammation in the liver through a stable mechanism that is not observed with acute exercise.^[^ ^27^, ^28^ ^]^ These data confirmed that EX‐R has a beneficial effect on NAFLD; however, for the first time, EX‐IR was observed to aggravate NAFLD, which raised the question of the relationship between exercise mode and exercise benefits for the body.

## 3 Discussion

Here, we report an exercise‐sensitive brain–liver circuit that links the effects of exercise on fear memory extinction in the brain and inflammation in the liver. Hippocampal and hepatic ACh levels are modulated by exercise. Exercise‐induced changes in ACh‐α7‐nAChR in hippocampal astrocytes not only regulate fear memory extinction but also influence hepatic ACh levels by affecting the amygdala–DMV–hepatic vagus circuits, thereby contributing to hepatic inflammation. In the brain, exercise—particularly regular exercise—significantly attenuates the persistence of fear memory. These results indicated that EX‐R not only enhances the extinction of fear memory but also inhibits its retrieval (Figure 1C). This effect is dependent on a reduction in axonal mRNA transport and local translation of ChAT in septal cholinergic projections to the hippocampus, which subsequently decreases the level of ACh and its activation of α7‐nAChR on astrocytes in the hippocampus. However, in terms of peripheral regulation, irregular exercise activates the amygdala–DMV–hepatic vagus circuit, increasing hepatic ACh levels and recruiting a novel FBXL6^high^ neutrophil subset, which promotes the interaction of these neutrophils with hepatocytes via Fe to upregulate the COX‐2/5‐LOX‐mediated metabolism of AA in hepatocytes, aggravating rather than alleviating NAFLD in mice. Taken together, for the first time, we report that exercise induces acetylcholine‐mediated regulation that connects brain function with peripheral hepatic function. Furthermore, we address the associated mechanisms that highlight the benefits of regular exercise and note the increased potential risks associated with irregular exercise. These findings may offer in‐depth molecular insights into the physiology and pathology of exercise conditions. Although a limited number of studies have reported the modulatory effects of exercise on fear memory,^[^ ^29^, ^30^, ^31^, ^32^, ^33^ ^]^ the underlying mechanisms through which cellular, molecular, and biochemical pathways are influenced remain poorly understood. Furthermore, clarity is lacking regarding whether and how these mechanisms are connected to brain–body interactions and overall bodily health. Here, for the first time, we demonstrate that both regular and irregular exercise, especially regular exercise, are beneficial for improving fear memory extinction, even though this modulation orchestrates exercise‐induced modulation in the liver.

The regulation of fear learning and extinction is mediated by cholinergic mechanisms. Cholinergic neurons in the basal forebrain transmit signals to both the neocortex and subcortical limbic regions, which include the hippocampus and amygdala.^[^ ^34^, ^35^, ^36^, ^37^ ^]^ Among them, inhibition of ACh‐α7‐nAChR signaling in hippocampal astrocytes has been verified to promote fear memory extinction.^[^ ^16^ ^]^ Our findings indicate that both regular and irregular exercise inhibit this mechanism by reducing the levels of ACh in the hippocampus through decreased axonal transport of ChAT mRNA from the medial septum (MS) to the hippocampus. Recent research has shown that compared with somatic transcripts, transcripts localized in the neuropil exhibit a greater preference for monosomes. This phenomenon may facilitate the production of a more diverse array of proteins from a limited pool of available ribosomes at synapses.^[^ ^38^, ^39^, ^40^ ^]^ This report presents the novel finding that ChAT is involved in axonal transport and local translation mechanisms. Furthermore, the results revealed that the axonal transport of ChAT mRNA relies on a complex formed by the RNA binding protein STRAP and the axon growth‐associated protein BASP1. These findings suggest potential novel modulatory effects on axonal transport and translation, which may be influenced not only by RNA‐binding proteins (RBPs) but also by axonal growth factors, such as BASP1. Further exploration of these interactions is warranted.

The liver, which is the main center of metabolism, is closely related to the brain under physiological and pathological conditions. Emerging evidence highlights the brain‒liver axis as a critical bidirectional pathway that regulates cognitive function and metabolic homeostasis. Chronic stress activates the locus coeruleus (LC) to serotonergic neurons in the rostral medullary raphe region (rMR) neural circuit, increasing sympathetic norepinephrine release in the liver and inhibiting hepatocyte proliferation, impairing liver regeneration.^[^ ^41^ ^]^ Conversely, dysregulated hepatic lipid metabolism (e.g., elevated cholesterol and low‐density lipoprotein levels) is linked to increased risks of neurodegenerative diseases such as amyotrophic lateral sclerosis (ALS) through systemic inflammation and disrupted blood‒brain barrier integrity.^[^ ^42^ ^]^ E. *et al.* reported that exercise intensities increase the ability of the liver to import lactate in mice, which is speculated to be favored over import by the brain.^[^ ^43^ ^]^ Wei *et al.* summarized the communication and ageing processes of the liver and brain and emphasized the metabolic mechanisms of the liver–brain axis through which exercise ameliorates ageing‐related neurodegenerative diseases.^[^ ^44^ ^]^ Here, our findings demonstrate that the amygdala–DMV–hepatic vagus circuits are activated during irregular exercise, contributing to an increase in hepatic ACh. However, the regular exercise‐mediated robust inhibitory effect on ACh‐α7‐nAChR signaling in hippocampal astrocytes is sufficient to abolish the excitation of the amygdala–DMV–hepatic vagus circuits. No significant increase in hepatic ACh was detected in the regular exercise group compared with the physically inactive group. These findings provide an inaugural demonstration that hippocampal ACh flux plays an essential role in regulating hepatic ACh flux, thereby offering novel insight into brain‒liver interactions. Previous studies on the relationship between ACh and exercise have focused primarily on its modulatory effects on skeletal muscle, cardiac function, and blood pressure.^[^ ^45^, ^46^ ^]^ However, investigations into the relationship between ACh and exercise within the neuronal system are limited. Exercise has been reported to increase ACh levels in the brain and improve spatial learning impairments.^[^ ^47^ ^]^ A potential mechanism may involve reduced acetylcholinesterase (AChE) activity in the medial prefrontal cortex (mPFC) and hippocampus (HC) as a result of exercise.^[^ ^48^ ^]^ However, whether exercise‐regulated ACh is sensitive to different modes of exercise remains unclear. In the present study, compared with irregular exercise, regular exercise significantly reduced the axonal transport of ChAT mRNA from the MS to the hippocampus by inhibiting the formation of the STRAP‐BASP1 complex. This reduction led to decreased ACh levels in the hippocampus of mice in the regular exercise group compared with those in the irregular exercise group, which subsequently resulted in more pronounced fear memory extinction in the regular exercise group. These findings indicate that different modes of exercise lead to distinct effects on ACh in the brain, ultimately resulting in varying outcomes in memory ability.

With respect to the association between ACh and liver diseases, some reports have shown that, in the liver, ACh and the activation of α7‐nAChR signaling in resident hepatic cells, such as hepatocytes, Kupffer cells, and satellite cells, inhibit inflammation and alleviate abnormal lipid metabolism in NAFLD.^[^ ^49^, ^50^, ^51^ ^]^ However, in this study, we reported that irregular exercise increased hepatic ACh levels, which was accompanied by the infiltration of a novel FBXL6^high^ neutrophil subset. Interestingly, FBXL6 interacted with α7‐nAChRs to switch from anti‐ to proinflammatory effects of ACh–α7‐nAChR signaling by blocking JAK‐STAT activation to activate NF‐κB in FBXL6^high^ neutrophils. Since FBXL6 was previously reported to promote p53 and KARS ubiquitination and degradation, which are associated with the occurrence of tumors in humans,^[^ ^52^, ^53^, ^54^ ^]^ we speculate that the inhibition of α7‐nAChR–JAK‐STAT signaling by FBXL6 results from its ability to promote the polyubiquitination and proteasomal degradation of these key proteins. In fact, this infiltrating FBXL6^high^ neutrophil subset not only presents a proinflammatory gene profile characterized by high expression of *Cxcl2*, *Il1b*, *Cd14*, *Fos*, and *Junb* but also significant expression of *Fth1*, all of which are target genes of the transcription factor NF‐κB. Consistent with these findings, the FBXL6^high^ neutrophil subset was rich in Fe with high expression of *Fth1*, and the released Fe could be absorbed by hepatocytes via TFRC. More importantly, the influx of Fe in hepatocytes increased the activity of COX‐2/5‐LOX, increasing AA metabolism in the liver. These findings support our findings that COX‐2/5‐LOX is activated by Fe.^[^ ^24^ ^]^ Our protein 3D structure bioinformatics analysis revealed that the enzyme COX‐2/5‐LOX required Fe as a cofactor, which confirmed the effect of the FBXL6^high^ neutrophil subset on the liver in the irregular exercise group.

This potential inflammatory risk to the liver induced by irregular exercise does not induce significant inflammatory damage in normal littermates. We speculate that the risk could be balanced or adjusted by the whole body if liver function is sufficiently healthy to modulate these hazard factors. However, pathological conditions, such as NAFLD, are not the same. Previously, many analyses have investigated the correlation between physical activity and disease recovery in patients with NAFLD, although various exercise regimens have been shown to affect liver fat content; there is no definitive evidence to recommend one regimen over another.^[^ ^55^, ^56^, ^57^ ^]^ Moreover, the impact of irregular exercise on NAFLD remains unexplored. Multiple findings have indicated that the metabolism of AA aggravates NAFLD.^[^ ^58^, ^59^, ^60^, ^61^ ^]^ Here, we found that irregular exercise induces the infiltration of the FBXL6^high^ neutrophil subset into the liver to increase the metabolism of AA, which does not affect liver function in normal mice; however, we strongly speculate that exercise is harmful to NAFLD mice. As expected, regular exercise significantly inhibited lipid deposits in NAFLD mice, which is consistent with previous reports. In contrast, compared with no exercise and regular exercise, EX‐IR significantly increased lipid deposits. An increase in ACh levels to activate AChRs on hepatocytes or resident hepatic cells, including Kupffer cells and satellite cells, has been reported to alleviate NAFLD and alcohol‐associated liver injury.^[^ ^62^, ^63^, ^64^ ^]^ In contrast, in this study, irregular exercise increased ACh levels and was accompanied by FBXL6‐mediated neutrophil infiltration such that FBXL6 interacted with α7‐nAChRs to switch downstream signaling from anti‐inflammatory to proinflammatory signaling, altering the effect of ACh on NAFLD. Some studies have shown that after intense exercise, immunity is suppressed, and individuals are susceptible to infection. Other studies have demonstrated that the inflammatory response is important for physiological adaptation after exercise training. The FBXL6^high^ neutrophil subset is a potential factor involved in these different exercise modes. These data describe, for the first time, the potential hazards and risks of irregular exercise, which has received little attention, and confirm that regular exercise is considered favorable for hepatic and cardiovascular health.

In this study, the exercise‐induced novel and specific links between the brain and liver were studied only in cell lines or animal models, and more cohort studies are needed for further validation. The modulation of the immune response by exercise depends on several factors, including regularity, intensity, duration, and type of exercise. Although we controlled the intensity, duration and type of exercise and designed a random frequency and interval time as irregular exercise, several other potential irregular exercise modes remained, and we did not verify the similar effects and mechanisms. In addition, we did not investigate why irregular exercise induced the infiltration of FBXL6^high^ neutrophils into the liver, which resulted in the heterogeneity of neutrophils after irregular exercise, and why this reprogramming of neutrophils depends on the hepatic, circulating or bone marrow environment. Nevertheless, by providing novel evidence and elucidating the underlying mechanisms, our results recommend regular exercise rather than irregular exercise for fitness in our daily life and in a variety of populations for athletic training and rehabilitative purposes.

## 4 Experimental Section

### Mouse Strain Generation and Breeding

C57BL/6 male mice aged between 8 and 10 weeks were obtained from Beijing Vital River Laboratory Animal Technology. The animals were kept in an environment with a 12‐h light‒dark cycle maintained at a temperature range of 22–25°C and 50%–60% relative humidity, with unrestricted access to food and water. All animal studies received approval from the Institutional Animal Care and Use Committee of the Army Medical University (No. AMUWE20228026). The C57BL/6J‐Chrna7^LOXP/LOXP^ variant was generated by GemPharmatech (Chengdu, China) as previously published.^[^ ^16^ ^]^ Ly6g‐Cre mice were purchased from Shanghai MODEL ORGANISMS. In addition, C57BL/6J‐ChAT‐Cre mice were acquired from another source. Loxp‐STOP‐Loxp‐Fbxl6 (LSL‐Fbxl6*^KI/+^*) mice were generated by Biocytogen Pharmaceuticals (Beijing, China) as previously published.^[^ ^52^ ^]^ LSL‐Fbxl6*^KI/+^* mice were crossed with Ly6g‐Cre mice to generate Fbxl6*^KI/+^* mice. MAP2‐Cre‐ERT2‐BASP1 mice were purchased from Shanghai MODEL ORGANISMS and induced with tamoxifen (Sigma‐Aldrich, CAS # 10540‐29‐1) dissolved in corn oil (Sigma‐Aldrich, CAS # 8001‐30‐7) for five consecutive days to generate BASP1‐KO mice. For the NAFLD model, the mice were randomly assigned to receive a diet designed to cause NAFLD, comprising 40% kcal from fat, 20% kcal from fructose, 2% cholesterol, and 0.5% cholic acid ad libitum. The mice were fed for 12 weeks beginning in the fourth week, and the final body weight reached more than 40 g.

### Chemicals and Antibodies

Anti‐ChAT (Abcam, ab224267, 1:100), anti‐α7‐nAChR (Abcam, ab216485, 1:500), anti‐GFAP (Abcam, ab302644, 1:250), anti‐STRAP (Invitrogen, PA5‐31226, 1:300), anti‐BASP1 (NOVUS, NBP2‐14347, 1:500), anti‐MAP2 (NOVUS, nb300‐213, 1:5000), anti‐p‐STAT3 (SYSY Antibodies, Cat. No. 226017, 1:1000), anti‐Ly6g (Abcam, ab25377, 1:300), anti‐FBXL6 (Bioss, bs‐16041R, 1:500), anti‐p‐STAT3 (Cell Technology, Cat. No. 9145T, 1:2000), anti‐p‐JAK2 (Cell Technology, Cat. No. 3776S, 1:1000), anti‐p‐IκBα (Cell Technology, Cat. No. 2859T, 1:1000), and anti‐GAPDH (Cell Technology, Cat. No. 5174T, 1:1000) antibodies were used. Goat anti‐rabbit IgG horseradish peroxidase (HRP) was purchased from BIOMIKY (Shanghai, China). Goat anti‐rabbit IgG H&L (Alexa Fluor^®^ 488), goat anti‐rabbit IgG H&L (Alexa Fluor^®^ 555), and goat anti‐rat IgG H&L (Alexa Fluor^®^ 555) conjugated antibodies were purchased from Abcam (USA).

### Cell Culture and Co‑culture System

Neutrophils were extracted from the bone marrow of the mice by the Tianjin Haoyang Biological Manufacture Company in China (Lot: LZS11131, LZS1100, TBD2013NR), following the manufacturer's guidelines.^[^ ^65^ ^]^ The purity of the isolated neutrophils exceeded 85%, as verified by fluorescence‐activated cell sorting (FACS) using a specific marker [mouse: CD45^+^, LY6G^+^]. The obtained neutrophils were diluted in RPMI‐1640 media (SH30027.01; HyClone, USA) supplemented with 10% (v/v) fetal bovine serum (FBS) (10,091,148; Gibco, USA) to a final concentration and cultured at 37 °C in an atmosphere of 5% (v/v) CO_2_.

Primary hepatocytes from mice were obtained from the specified groups via the liver perfusion technique outlined in previous studies.^[^ ^52^ ^]^ In brief, an incision was made in the abdominal cavity of each mouse under anesthesia to minimize discomfort. The liver tissue was then carefully perfused with 1× liver perfusion medium (#17701‐038; Gibco) and 1× liver digestion medium (#17703‐034; Gibco) through the portal vein. Afterward, 100 µm steel mesh was used to grind and filter the digested liver material. The primary hepatocytes were collected through centrifugation of the filtered solution at 800 rpm for 5 min at 4 °C and subsequently purified with 50% Percoll solution (#17‐0891‐01; GE Healthcare Life Sciences). The acquired hepatocytes were cultured in Dulbecco's modified Eagle's medium (DMEM) supplemented with 10% FBS and 1% penicillin‒streptomycin and maintained at 37 °C in a cell incubator containing 5% CO_2_. NG108‐15 cholinergic neurons were purchased from Fu Heng Biology (Shanghai, China), and the neurons were confirmed through immunostaining with a MAP2 antibody (NB300‐213; Novus, USA). All the cultured cells were maintained at 37 °C in a humidified incubator supplemented with 5% CO_2_. Primary isolated neutrophils were cultured with primary hepatocytes in six‐well plates for coculture. Hepatocyte neutrophils were cultured in DMEM supplemented with 10% FBS and 1% penicillin‒streptomycin and maintained at 37 °C in a cell incubator containing 5% CO_2_.

### Treadmill Exercise Protocol

The exercise protocol involved 60 min of forced running on a treadmill set at a 0% incline (Columbus Instruments). Prior to the experiment, the mice underwent two brief training sessions. The mice were randomly divided into two groups: for the regular exercise group (EX‐R), the mice were subjected to a total of 12 exercises three times a week for four weeks (one Monday, one Wednesday, and one Friday), whereas for the irregular exercise group (EX‐IR), the total amount of 12 exercises was distributed irregularly to four weeks according to the number of durations and intervals shown in Figure 1A. The single‐treadmill regimen starts at a warm‐up speed of 5 m min^−1^ for 5 min. The speed was then increased by 5 m min^−1^ every 5 min until it reached a maximum speed of 20 m min^−1^, with a total running time of up to 1 h occurring between 9:00 and 11:00 am. The sedentary control mice were brought to the training room daily before the exercise groups, and while in their home cage, they were exposed to the treadmill turned on in the background to ensure the same stress adaptation to the noise for all the mice. Moreover, sedentary mice were restrained and manipulated daily to expose them to a level of human interaction/handling comparable to that of their exercised counterparts.

To estimate maximal exercise capacity, a VO_2_‐max treadmill test was performed using a ramp protocol that maximized running within 15 min at a 20° incline. The power output in watts was calculated on the basis of the incline per minute and the weight of the mice.

### Metabolic Cage Studies

To evaluate various metabolic parameters in the mice, CLAMS (Columbus Instruments) metabolic cages were utilized. In brief, the mice were placed in individual metabolic cages without bedding and with unrestricted access to a standard chow diet and tap water and allowed to acclimate for 72 h. The mice underwent noninvasive daily monitoring of gas exchange, physical activity, and food intake, which was measured via a balance connected to each CLAMS cage. An Echo‐MRI 3‐in‐1 Body Composition Analyser (Echo Medical Systems) was used to assess total body fat and lean mass. The respiratory exchange ratio (RER) was calculated as the ratio of carbon dioxide produced to oxygen consumed, whereas energy expenditure (kcal/h) was derived from gas exchange data. Physical activity was measured by counting beam breaks within a grid of photosensors integrated into the cages. Total activity was defined as the overall number of beam breaks, whereas ambulatory activity was determined by consecutive beam breaks within the grid.

### Fear Conditioning and Behavior

An associative fear conditioning paradigm was used in which a CS (an auditory cue) was paired with an unconditioned aversive stimulus (a footshock), as published previously.^[^ ^16^ ^]^ The CS consisted of 9.9‐s sequences of pure tone (8 kHz, 70 dB sound pressure level), made up of 33 square pulses, each lasting 50 ms, with an interpulse interval of 250 ms. The unconditioned stimulus was a 1‐s footshock (0.6–1 mA), which was administered simultaneously with the conclusion of the final sound pulse. There was a 3‐min interval between each pair. For experiments conducted during wakefulness, a custom‐designed cage was utilized, measuring 30 × 20 × 20 cm, featuring stainless steel shock grids linked to a feedback current‐regulated shocker (conditioning box). A charge‐coupled device (CCD) camera operating at 30 Hz with infrared lighting and an electrostatic speaker were positioned on the top and sidewalls of the box, respectively. Prior to the fear conditioning day, the experimenter gently handled the mice for 5 min each day over a span of 3 d. On the conditioning day, the mice were allowed to acclimate to the shocking grids for 5–10 min before the pairings commenced. Three‐ to five‐way pairings were administered at 3‐min intervals to facilitate fear conditioning. The mice were returned to their home cages 5 min post‐pairing. One day after conditioning, the mice underwent either fear retrieval for fiber recordings or behavioral assessments in a different context with nonshocking grids. All the equipment was thoroughly cleaned with ethanol and rinsed with water between sessions.

An open‐field test (OFT) was performed to evaluate the general exploratory locomotion of the mice. Before the test, the animals were placed in the laboratory for 2 h to help them adapt to the environment. During the experiment, the mice were placed in the central area of an opaque open‐field apparatus and allowed to freely explore it for 5 min. The total distance travelled was automatically recorded for the analysis (ANY‐maze; Stoelting Co., Ltd., Wood Dale, IL). To remove olfactory cues, the apparatus was cleaned with 70% ethanol between each trial.

In the Y‐maze test specifically, a Y‐maze made of grey polyvinylidene was placed in a quiet and illuminated room. Each maze contained three arms (8 × 30 × 15 cm, width × length × height), with an angle of 120° between each arm. The three arms of the Y maze were randomly set to A, B, and C, and the mouse wall was placed in the initial area for 5 min to adapt to the experimental environment. The mouse was placed again at the end of one arm and allowed to explore freely for 8 min, and the correct number of alternations among the three different arms, such as ABC, ACB, BAC, BCA, CAB, and CBA, was recorded. From this, the total number of times each arm was entered and the total number of alternations were counted, and the spontaneous alternating reaction rate (%) = [number of correct alternating reactions/(*N*−2)] × 100 was calculated according to the formula.

### Introduction of a Virus to Facilitate GCaMP6f Expression

C57BL/6 mice were subjected to anesthesia via isoflurane (1%–1.5% in pure O_2_) and positioned within a stereotaxic apparatus following established protocols. A small vertical incision was made in the skin, and the skull above the targeted injection site was carefully thinned to facilitate the insertion of a glass micropipette filled with pAAV. AAV constructs, specifically AAV5‐GfaABC1D‐cytoGCaMP6f‐SV40, were administered at a volume of 800 nL per site at no dilution. These AAV vectors were regulated by astrocyte‐specific sGFAP or the shorter 681‐bp GfaABC1D promoter. The injection was performed with a micropipette attached to a micromanipulator (H. Saur) mounted on a custom platform, delivering a total of 150–300 nL of the viral construct at different depths in increments of 100 µm at a rate of 0.1 µL min^−1^. Following the injection, the pipette was maintained in position for 15 min before being gradually withdrawn from the brain. The incision in the scalp was sealed with tissue adhesive (Vetbond; 3M Animal Care Products), and analgesics were administered postinjection to support recovery. Optical fiber‐based recordings were conducted ≈20 d after viral injection, with GCaMP6 injection sites selected on the basis of experimental objectives: the dHP at coordinates AP −1.70 mm, ML ± 1.80 mm, and DV −2 mm and the MS at coordinates AP +0.75 mm, ML 0.00 mm, and DV −3.75 mm. For anterograde tracing, a 1 µL injection of rAAV‐hSyn‐DIO‐EGFP‐WPRE‐pA (UNC Vector Core) was administered in the MS, with additional coordinates provided for the dHP (AP −1.70 mm, ML ± 1.90 mm, DV −1.70 mm) and the MS (AP −0.8 mm, ML ± 0 mm, DV −3.6 mm).

### Optic Fiber‐Based Recordings in Freely Moving Mice

In the procedure for fiber implantation surgery, mice expressing GCaMP6f were anaesthetized with isoflurane and secured in a stereotaxic head frame. An optical fiber measuring 200 µm in diameter with a numerical aperture of 0.48 (Catalogue No. MFP_200/230/900‐0.48; Doric) was carefully inserted at the site of pAAV injection and subsequently affixed to the skull with dental cement (Tetric EvoFlow; Ivoclar Vivadent Corporation). For experimental purposes, the ends of the optical fibers were positioned in the dHP at the coordinates AP −1.70 mm, ML ± 1.90 mm, and DV −1.7 mm. Calcium ion (Ca^2+^) recordings were conducted using a modular fiberoptic device, as previously described. The fluorescence signals were captured at a sampling rate of 2000 Hz through custom software developed in LabVIEW. Concurrently, animal behavior was recorded with a camera operating at 30 Hz under infrared light. The optical fiber recordings commenced after a three‐day acclimatization period, during which the experimenter handled the animals for five minutes each day. On the recording days, the mice were allowed to habituate to the shock cage for 5–10 min prior to the experiments. After the completion of the fiber recording sessions, the mice were euthanized for histological analysis. Following perfusion with 4% paraformaldehyde (PFA) and overnight dehydration in 15% sucrose, the brains were sectioned into 50‐µm slices for imaging using either a confocal microscope (LSM 700; Zeiss) or a stereoscope (Olympus).

### Histology and Confocal Imaging

The mice were subjected to anesthesia with ketamine and subsequently perfused with ice‐cold 4% PFA in phosphate‐buffered saline (PBS). The brains were extracted and immersed in 4% PFA overnight before being transferred to a 30% sucrose solution for 1–2 d until they were completely submerged. Using a microtome, the brains were sectioned into 40 µm thick slices and preserved in an antifreeze solution at −20 °C for future experiments. For the staining of floating sections, the brain slices were rinsed twice with PBS, followed by a 20‐min permeabilization step using 0.5% Triton‐100 in Tris‐buffered saline (TBS). After a 5‐min wash with TBS+ (TBS containing 0.05% Triton‐100) and a 30‐min blocking period with 3.5% donkey serum in TBS+, the sections were incubated overnight at 4°C with the primary antibody while shaking. The following day, the brain sections were washed with TBS+ and then incubated with the secondary antibody at room temperature for 2 h.

Confocal imaging was performed with an Olympus FLUOVIEW1000 confocal microscope utilizing a 40× oil objective (NA1.30) with an XY resolution of 0.4975 µm pixel^−1^ and a Z resolution of either 1.0 or 1.5 µm per slice. Tiled images were captured according to the locations of the fluorescent signals, and the images were subsequently stitched with Olympus FluoView imaging software. Adjustments to brightness and contrast were made with ImageJ.

### AAV‐Mediated Astrocytic nAChR Deletion in the dHP

LoxP‐Chrna7 transgenic mice, referred to as Chrna7^loxP/loxP^, were injected with rAAV‐GfaABC1D‐NLS‐Cre‐P2A‐mCherry, a plasmid generously provided by B. Khakh (Addgene plasmid no. 105603; Research Resource Identifier: Addgene_105603). This injection was administered into the dHP at the coordinates AP −1.70 mm, ML ±1.90 mm, and DV −1.70 mm. The AAV vectors utilized were specifically designed to be driven by the astrocyte‐specific GfaABC1D promoter, facilitating the excision of loxP sites through Cre recombination. Three weeks after virus injection, behavioral experiments were conducted with these mice.

### Metabolomics

Metabolomic analysis was conducted according to established protocols involving the extraction of small biochemicals from six independent brain and liver samples per experimental group via methanol. These extracts were subsequently analyzed through ultrahigh‐performance liquid chromatography‒tandem mass spectrometry (UPLC‐MS/MS) in both positive and negative modes, as well as gas chromatography‒mass spectrometry (GC‐MS). Metabolite identification was achieved by automatically comparing the ion features, which included parameters such as retention time, molecular weight (m/z), preferred adducts, in‐source fragments, and corresponding MS spectra, from the experimental samples against a reference library of chemical standards (BGI‐Shenzhen, China).

### Transcriptomics

Total RNA extraction from the livers was carried out via TRIzol (Life Technologies) and Direct‐zol RNA MiniPrep (Zymo Research Corporation), following the protocols provided by the manufacturers. For library preparation, a TruSeq‐stranded mRNA sample preparation kit (Illumina) was used in accordance with the manufacturer's guidelines. Sequencing was conducted on the Illumina NOVASeq 6000 platform in paired‐end mode with a read length of 100 bp (BGI‐Shenzhen, China).

### scRNA‐seq

Immune cells were isolated from peripheral blood via a high‐density Ficoll gradient. Briefly, peripheral blood was diluted tenfold with FACS buffer (2% FBS in PBS), carefully layered on a Ficoll gradient (11191; Sigma, Germany) and centrifuged at 400× *g* for 30 min at room temperature. The buffy coat was carefully removed, diluted fivefold with FACS buffer, pelleted (300× *g*, 5 min, 4 °C), and incubated in cold FACS buffer containing DNase I (LS006344; Worthington Biochemical Corporation, USA) for 10 min at 4 °C. Clumps were dispersed into a single‐cell suspension by gentle pipetting. Single‐cell suspensions were prepared and loaded on a Chromium Controller (10× Genomics, Pleasanton, CA) following the manufacturer's specifications. The Chromium Controller and Chromium Single‐Cell 3′ Reagent Version 2 Kit (10× Genomics, Pleasanton, CA) was used to construct a single‐cell library through 10× scRNA‐seq via the DNBSEQ platform (BGI‐Shenzhen, China). The gene count matrix generated with Cell Ranger v7.1.0 was converted into a data format (AnnData) compatible with the Scanpy v1.9.3 platform.^[^ ^66^ ^]^ Doublets were removed via Scrublet v0.2.1 using the default parameters. Principal component analysis was performed for dimensionality reduction with the scanpytl.pca function (svd_solver = “arpack”).^[^ ^67^ ^]^ Uniform manifold approximation and projection were then used for two‐dimensional visualization of the resulting clusters. The marker genes of each cluster were identified with the scanpy rank_genes_groups function (method = “Wilcoxon”), and the clusters were annotated by a cell typist. DEGs were annotated via Gene Ontology (GO) enrichment analyses.

### Western Blot Quantification and Coprecipitation

Total protein was extracted using 2% sodium dodecyl sulfate (SDS) buffer containing a protease inhibitor and a phosphatase inhibitor cocktail (Solarbio, BC3710). Protein (5–20 µg) was separated by SDS‐PAGE and then transferred to a 0.22 µm polyvinylidene fluoride membrane using the semidry transfer method. The membrane was blocked with 5% fat‐free milk in TBST (Tris‐buffered saline with 0.1% Tween‐20) for 1 h at room temperature and then incubated with a primary antibody diluted in blocking solution overnight at 4 °C. The membrane was then washed in TBST (5 × 7 min) and incubated with species‐specific HRP‐conjugated secondary antibodies for 1 h at room temperature. After five TBST washes (7 min), protein bands were visualized using chemiluminescence (ECL) Western blotting substrate (Pierce) and imaged with a ChemiDoc Touch Imaging System. Quantitative analysis of protein levels was conducted using ImageJ (version 1.8.0). Co‐IP was conducted with a Pierce Crosstalk Magnetic IP/Co‐IP Kit (88,805; Thermo Fisher Scientific, USA). Neutrophils were isolated from a total of 1 × 10^8^ cells and lysed in lysis buffer containing a protease/phosphatase inhibitor cocktail (Pierce) for 30 min on ice. The lysate was subsequently collected through high‐speed centrifugation at 13 000× *g* for 10 min at 4 °C. The supernatant was then incubated with FBXL6 antibody, which was cross‐linked to protein A/G magnetic beads, and allowed to incubate overnight in a refrigerator at 4 °C. Following washing with IP lysis buffer, the resulting products were analyzed via Western blotting.

### RNA Pull‐Down

The RNA pull‐down assay was conducted following the guidelines provided by the manufacturer (Sangon Biotech, Shanghai, China). In summary, biotinylated sense and antisense probes were synthesized directly utilizing the ChAT gene sequences from Sangon Biotech (Shanghai, China) as follows: ChAT‐Sense‐F, TAATACGACTCACTATAGGG TCTAGCTGTGAGGAGGTGCT; ChAT‐Sense‐R, CTTGGTTGGGCCTCTAGCTC; ChAT‐Antisense‐F, TCTAGCTGTGAGGAGGTG CT; and ChAT‐Antisense‐R, TAATACGACTCACTATAGGG CTTGGTTGGGCCT CTAGCTC. Mouse brain tissue lysates were obtained, and Pierce nucleic acid‐compatible streptavidin magnetic beads were added to the labelled biotin‐RNAs. The samples were incubated and subjected to the pull‐down protocol, and the protein was eluted from the column. Finally, the eluted proteins were analyzed via mass spectrometry and identified via proteomic sequencing.

### RNAscope and Immunofluorescence

Fluorescence in situ hybridization was conducted according to previously established protocols,^[^ ^68^ ^]^ with some adjustments. In brief, fixed neurons were rinsed in PBS supplemented with 5 × 10^−3^ m MgCl_2_ and equilibrated in 1× SSC buffer for 10 min. The cells were subsequently washed in 10% formamide (Sigma‐Aldrich) for an additional 10 min before being preincubated in hybridization buffer, which consisted of 20% dextran sulfate, 4× SSC, 4 mg mL^−1^ bovine serum albumin (BSA), 20 × 10^−3^ m ribonucleoside vanadyl complex, and 10 × 10^−3^ m sodium phosphate buffer at pH 7.0, at 37 °C for 1.5 h. Probes (1 µL) were mixed in 50 µL of hybridization buffer and incubated with coverslips at 37 °C overnight. The Stellaris FISH probes, which were directly labeled with Quasar570, were sourced from Biosearch Technologies. In addition, a Cy3‐labelled oligo dT probe (Biosearch Technologies) was utilized to identify poly(A)‐positive mRNAs. The specificity of the probes was validated via the use of a GFP control probe. For the immunofluorescence assays, fixed motor neurons were incubated overnight at 4 °C with antibodies from Epitomics (1:250) and BD Biosciences (1:500) in blocking buffer containing 5% BSA and 1× PBS. Cy3‐conjugated secondary antibodies (Jackson ImmunoResearch) were applied for 1 h at room temperature.

### Fe Assay and ELISA Experiments

For the detection of total intracellular iron or ferrous (Fe^2+^) levels in neutrophils or hepatocytes, cells and iron were collected. Iron concentration was measured using the Cell Total Iron Colorimetric Assay Kit (E‐BC‐K880‐M; Elabscience, China) and the Cell Ferrous Iron (Fe^2+^) Fluorometric Assay Kit (E‐BC‐F101; Elabscience, China), adhering strictly to the manufacturer's instructions. The quantification of iron and Fe^2+^ reduction in the presence of the chromogen was performed by measuring the absorbance at 590 nm with a spectrophotometer (Thermo Fisher, USA). For the acetylcholine assays, the brain or liver tissue of the sacrificed mice was isolated at low temperature as soon as possible, weighed and centrifuged to obtain the supernatant. A mouse ACh ELISA kit (Jingmei, Shenzhen, China), which was used in strict accordance with the reagent protocol, was used for ACh detection.

### siRNA and Quantitative Real‑Time PCR (qRT‑PCR)

All siRNAs were obtained from Wuhan GeneCreate Biological Engineering Co., Ltd. The specific siRNA sequences targeting the genes of interest are listed in **Table** **3**. The transfection was performed using Lipofectamine 3000 Transfection Reagent (Invitrogen, L3000001) in Opti‐MEM medium (Gibco) according to the manufacturer's protocol. The final concentration of the siRNAs was 50 × 10^−9^ m. Following transfection for 6 h, the medium was diluted in EME‐α supplemented with 20% (v/v) FBS, resulting in the isolation of neutrophils at a concentration of 1 × 107 cells and hepatocytes at a concentration of 1 × 10^6^ cells. In addition, for the knockdown of the FBXL6 gene in vivo, adult male C57BL/6 mice (8–10 weeks old) received injections of siRNA (50–100 µg in 100 µL of RNase‐free PBS/saline) or scrambled siRNA control via the lateral tail vein, followed by tail disinfection and vasodilation under brief heat. Injections were performed 1–2 h prior to treadmill exercise.

**Table 3:** siRNA sequences for the target genes.

Gene (siRNA)	Sense (5′–3′)	Antisense (5′–3′)
si‐NC	UUCUCCGAACGUGUCACGUTT	ACGUGACACGUUCGGAGAATT
si‐Chrna7‐1	GAAUGAGAAGUUCUAUGAA	UUCAUAGAACUUCUCAUUC
si‐Chrna7‐2	CUAUAACAGUGCAGAUGAA	UUCAUCUGCACUGUUAUAG
si‐Chrna7‐3	GGCCGGCUUGUCAGCACAA	UUGUGCUGACACCCGCC
si‐Fbxl6‐1	GCUCACCUUCCUCAAGCUU	AAGCUUGAGGAAGGUGAGC
si‐Fbxl6‐2	GCACUGGCAUGAACUGCAA	UUGCAGUUCAUGCCAGUGC
si‐Fbxl6‐3	GCGGCUGCUGAAUCUGAUU	AAUCAGAUUCAGCAGCCGC
si‐Fpn‐1	GCAGAUUAGCAGACAUGAA	UUCAUGUCUGCUAAUCUGC
si‐Fpn‐2	GAAUACAUCUGUUGUAUAA	UUAUACAACAGAUGUAUUC
si‐Fpn‐3	GACCGUCUUUACAACAGAA	UUCUGUUGUAAAGACGGUC
si‐Tfrc‐1	CCAGACCGUUAUGUUGUAGUA	UACUACAACAUAACGGUCUGG
si‐Tfrc‐2	CGUAUUAUGAAAGUGGAGUAU	UAGUCCAGGUUCAAUUCAACG
si‐Tfrc‐3	CGUAUUAUGAAAGUGGAGUAU	AUACUCCACUUUCAUAAUACG
si‐Fbxl6‐ESC	GAGUGGCUUAUGCCCAAUC	GAUUGGGCAUAAGCCACUCCA

Following being washed with ice‐cold PBS, the cells were directly lysed in TRIzol Reagent (Thermo Fisher, Cat# 15596026). Total RNA extraction was performed using the RNeasy Plus Mini Kit (Qiagen, Cat# 73404). cDNA was subsequently synthesized with a reverse transcriptase kit (Takara, Cat# DRR047S), with all isolation and reverse transcription steps strictly adhering to the manufacturers’ instructions. Quantitative real‐time PCR (qPCR) was conducted using FastStart Universal SYBR Green Master Mix (Roche, Cat# 04707516001) on a Roche Real‐Time PCR Detection System. Target gene expression levels were quantified using the 2^−^ΔΔCt method relative to those of the β‐actin housekeeping gene. The final results represent the fold change relative to that of the control samples. The primers for the Fbxl6 gene for qRT‑PCR were F‐TCTGGTTGGGGAGACCGTAT and R‐TCTGGAGCTGAGAGAAC CGA.

### Oil Red O Staining

The procedure for Oil Red O staining involved the fixation of HepG2 cells in a 4% PFA solution, followed by a 15‐min staining period with Oil Red O. After staining, the samples underwent a brief wash in 60% isopropanol, which was followed by three washes with PBS. The regions with positive staining results were analyzed using ImageJ software from the National Institutes of Health in Bethesda, MD.

### Molecular Docking

The α7‐nAChR/COX‐2/LOX‐5 crystal structure is based on the PDB code 7EKI/3LN0/6N2W. The structure of FBXL6/STRAP/BASP1 was predicted by the AlphaFold protein structure database code AF‐Q9QXW0‐F1‐v4/AF‐Q9Z1Z2‐F1‐v4/AF‐Q91XV3‐F1‐v4. Protein docking was performed by the GRAMM docking server, and the binding interactions between Fe^2+^ and proteins were determined by the CB‐Dock2 server. All the docking results were visualized by PyMOL software.

### Statistical Analysis

All the data were analyzed and presented via GraphPad Prism software (version 8). Sample sizes (*n*) denote biological replicates and are specified in the figure legends. For comparisons between two groups, two‐tailed unpaired *t*‐tests were used; for comparisons of ≥3 groups, one‐way ANOVA with Tukey's post hoc test was applied. Outliers were excluded via Grubbs’ test (α = 0.05). The data are presented as the mean±SEM; *P < 0.05, **P < 0.01, and ***P < 0.001; n.s., no significant difference.

## Conflict of Interest

The authors declare no conflict of interest.

## Author Contributions

P.M., K.Z., and L.Y. contributed equally to this work. M.‐T. M. and S.‐S. D. designed the research; P.M., K.Z., L.Y., C.Y., H.L., J.S., J.L., Q.B., and Y.H. performed the research; S.‐S.D., P.M., and K.Z. contributed analytical tools; S.‐S. D., P.M., K.Z., and M.‐T. M. analyzed the data and wrote the paper.

## Supporting information



Supporting Information

## Data Availability

The data that support the findings of this study are available from the corresponding author upon reasonable request.
