# Supplementary material for: Regular versus Irregular Exercise Differentially Modulates Hippocampal‐Hepatic Acetylcholine Flux to Coordinate Fear Memory Extinction and Liver Inflammation
Source: Adv Sci (Weinh). 2025 Sep 26;12(45):e00177. doi: 10.1002/advs.202500177 (PMC12677590; doi:10.1002/advs.202500177)
Supplement: Supplementary file 1 — Supporting Information [file ADVS-12-e00177-s001.pdf]

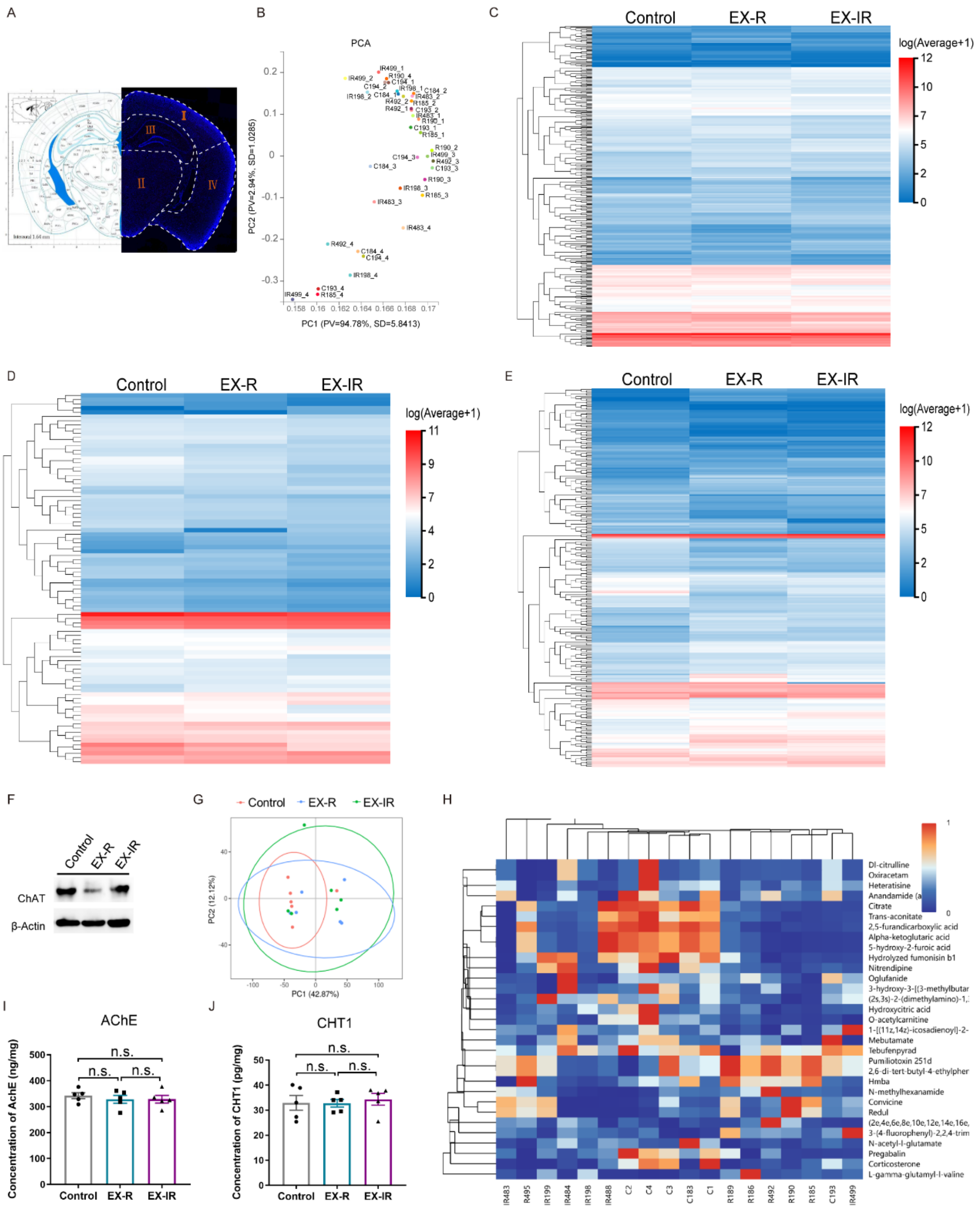

Figure S1. Transcriptome sequencing of other brain regions, including the cortex and thalamus, indicates that there are no differences in the expression of ChAT.

(A) Partition scheme for transcriptome sequencing of brain tissue.

(B) Transcriptome sequencing sample distribution. Each dot represents a sample.

(C) Heatmap of DEGs in area I from three different groups (n =3 mice). The red denotes expression of upregulated DEGs, the blue denotes expression of the downregulated

DEGs.

(D) Heatmap of DEGs in area II from three different groups (n =3 mice). The red denotes expression of upregulated DEGs, the blue denotes expression of the downregulated DEGs.

(E) Heatmap of DEGs in area IV from three different groups (n =3 mice). The red denotes expression of upregulated DEGs, the blue denotes expression of the downregulated DEGs.

(F) Western blot detection of ChAT protein in the hippocampus.

(G)Distribution of brain tissue metabolomics sequencing samples. Each dot represents a sample, as shown in the illustration, different colors indicate the group.

(H) Heat map of differential metabolites (n = 6 in each group).

(I) ELISA detection of AChE and CHT1 (J) in the hippocampus.

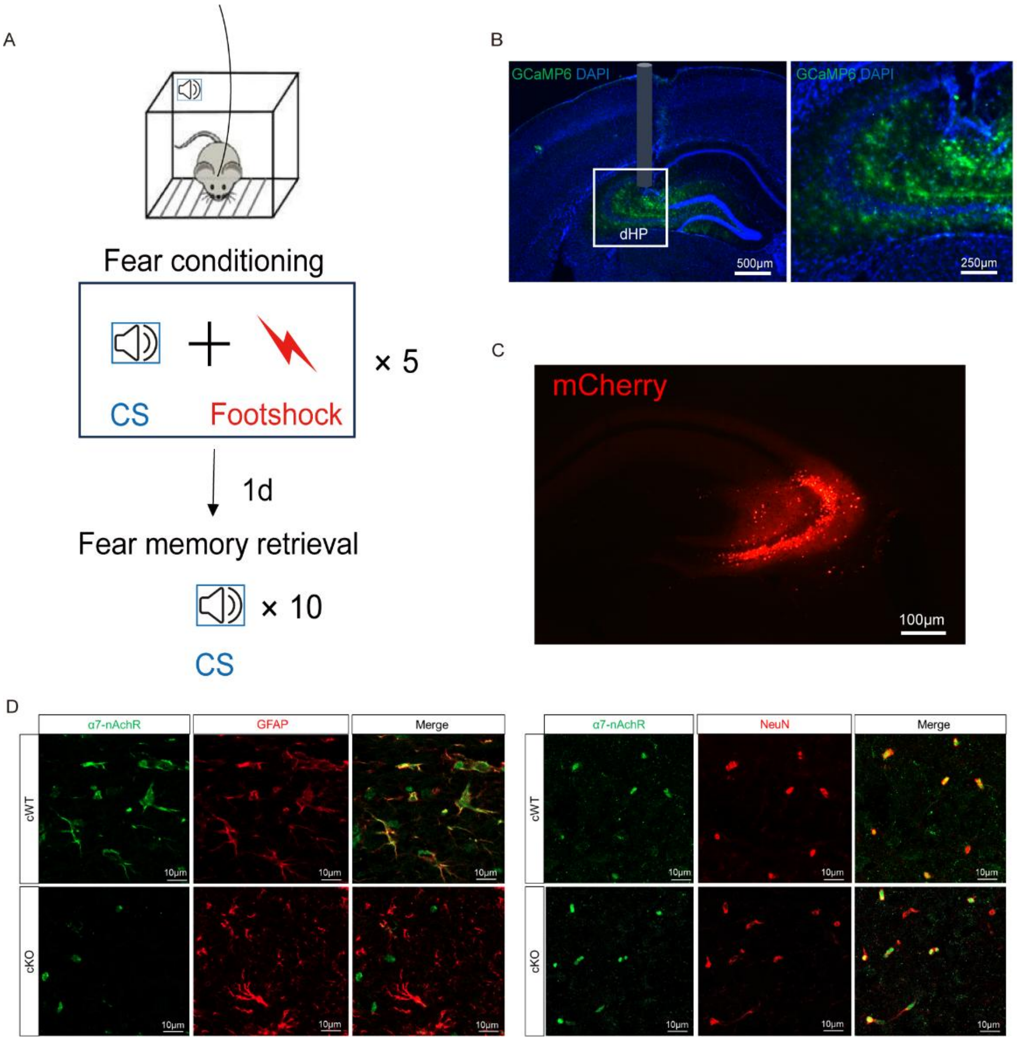

Figure S2. Behavioral validation with optic fiber and α7-nAchR cKO mouse construction.

(A) Top: Schematic of optical fiber recording. Bottom: Fear conditioning protocol

(B) Fluorescence image of astrocytes labelled with AAV5-GfaABC1D-cytoGCaMP6f-SV40 (green) and implanted optical fibers in the hippocampus.

(C) Confocal images of astrocytes labelled with rAAV-GfaABC1D-NLS-Cre-P2A-mCherry (red) in the hippocampus. rAAV-GfaABC1D-NLS-Cre-P2A-mCherry was injected into the hippocampus of Chrna7$^{loxP/loxP}$ mice to generate α7-nAchR cKO mice.

(D) Immunostaining of α7-nAchRs and astrocytes (GFAP)/neurons (NeuN) in the hippocampus of α7-nAchR cKO and cWT mice to verify that the α7-nAchR cKO mice were constructed successfully.

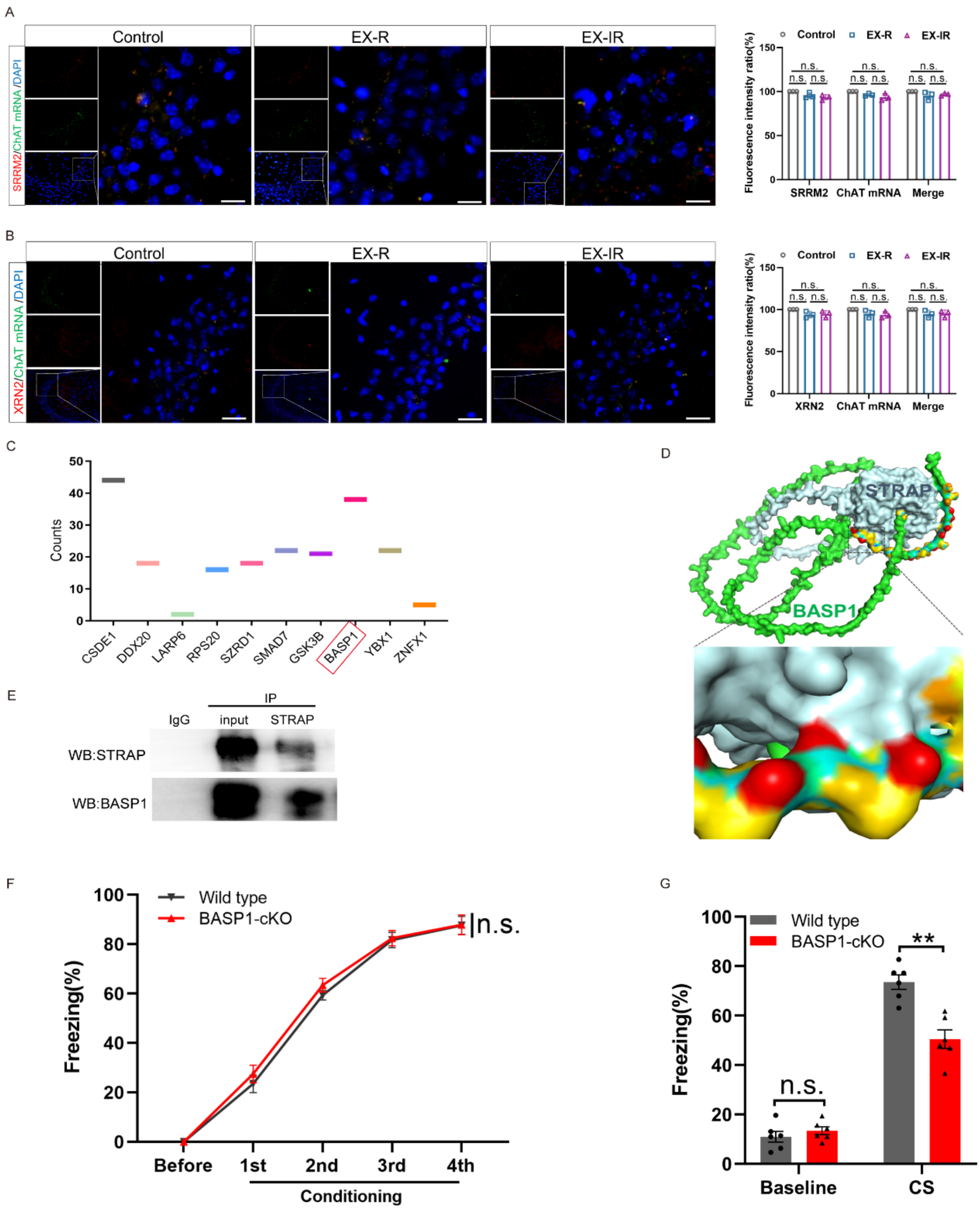

Figure S3. Functional detection of STRAP-BASP1 interaction.

(A) Immunostaining of SRRM2 and ChAT mRNA in the hippocampus; scale bar, 10 μm.

(B) Immunostaining of XRN2 and ChAT mRNA in the hippocampus; scale bar, 10 μm.

(C) Statistical plot of the mass spectrometry results.

(D) Visualization of the interaction between STRAP and BASP1 using PyMOL

software.

(E) Co-IP of STRAP and BASP1 from brain tissue.

(F) Freezing levels of BASP1-cKO mice during pairing.

(G) Freezing levels of BASP1-cKO mice at 1 day after retrieval.

The data are presented as the mean ± SEM. SEM, standard error of the mean. *P<0.05, **P<0.01, ***P<0.001. n.s., no significant difference.

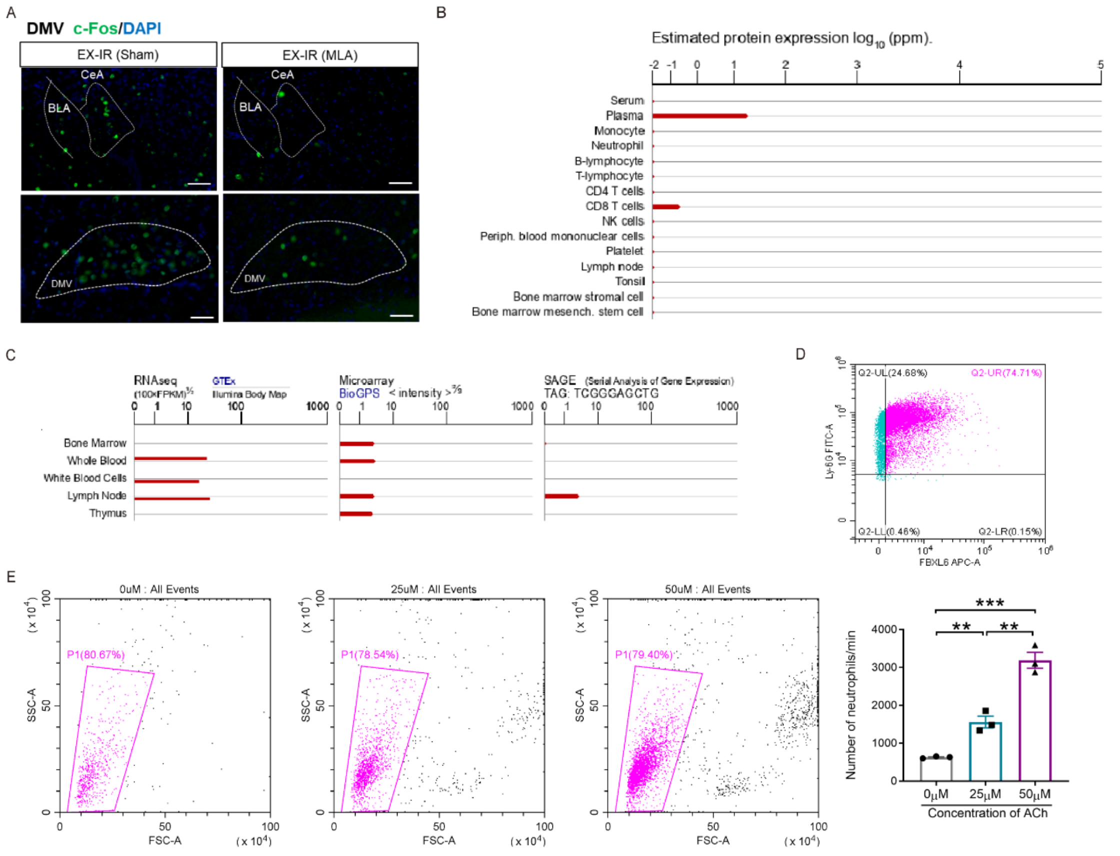

Figure S4. The database indicated that FBXL6 was barely expressed in normal neutrophils from either humans or mice.

(A) Fluorescence of cFos^{+} in the CeA and DMV of injected MLA/sham mice. Scale bar, 50 μm.

(B) GTEx, BioGPS, and SAGE analyses revealed that FBXL6 was barely expressed in human neutrophils.

(C) BioGPS data indicating that FBXL6 was barely expressed in mouse neutrophils.

(D) Flow cytometry analysis of FBXL6^{high} neutrophils.

(E) An in vitro Transwell assay was used to determine the number of FBXL6^{high} neutrophils in response to ACh. The data are presented as the mean ± SEM. SEM, standard error of the mean. *P<0.05, **P<0.01, ***P<0.001. n.s., no significant difference.

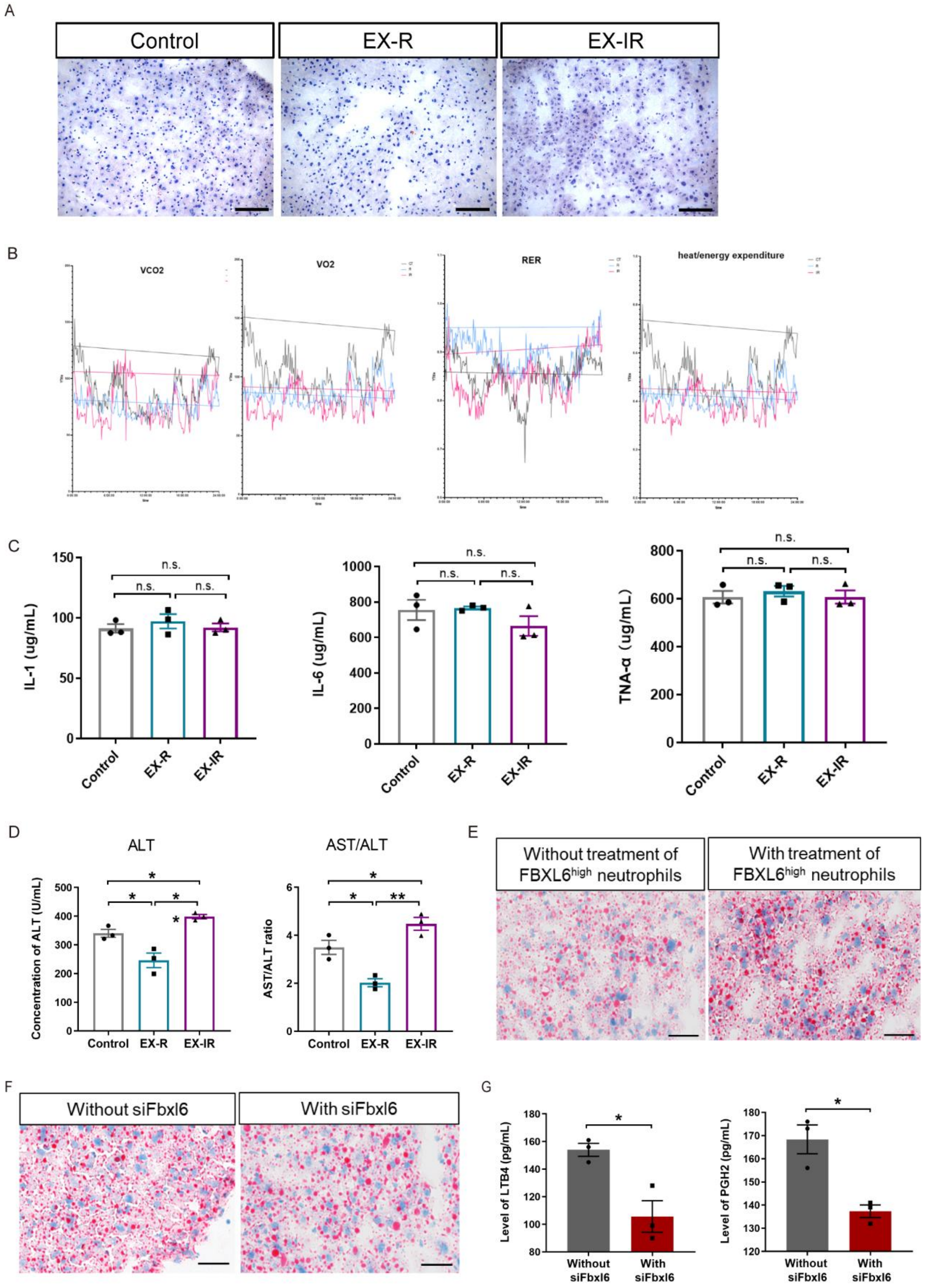

Figure S5. Basic metabolic indexes, liver oil red staining and inflammatory cytokine levels in mice.

(A) Oil red staining of the liver.

(B) Data statistics of the basal metabolic cages.

(C) Detection of inflammatory cytokines in the liver by ELISA.

(D) ALT levels and AST/ALT ratios in the blood of NAFLD mice after exercise.

(E) Lipid deposition in NAFLD mice injected with or not injected with $FBXL6^{high}$ neutrophils; scale bar, 50 μm.

(F) Lipid deposition in NAFLD mice with or without siFBXL6; scale bar, 50 μm.

(G) Concentrations of LTB4 and PGH2 in NAFLD mouse livers from mice treated with siFBXL6 or no siFBXL6; scale bar, 50 μm.

The data are presented as the mean ± SEM. SEM, standard error of the mean. *P<0.05, **P<0.01, ***P<0.001. n.s., no significant difference.

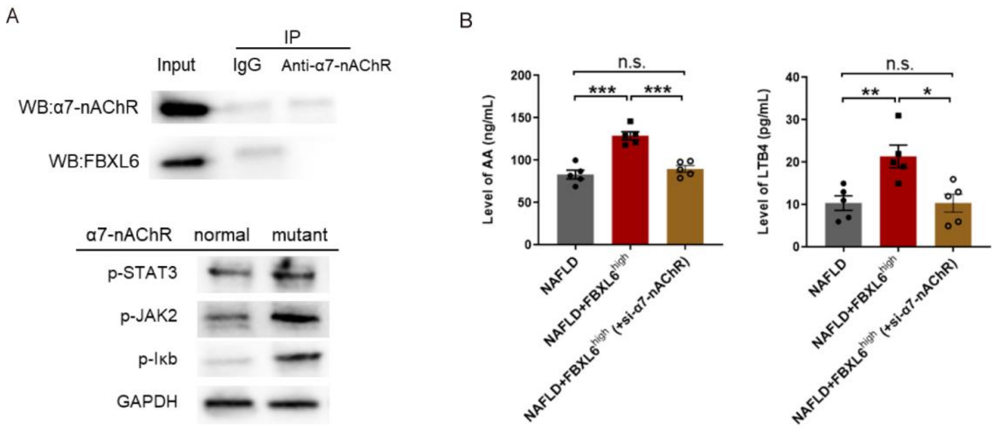

Figure S6. siRNA and truncated α7-nAChR to detect its function

(A) ELISA was used to detect the contents of AA and LTB4 after in vitro coculture.

(B) Western blot analysis of the interaction between the truncated FBXL6 protein and the activated downstream protein.

The data are presented as the mean ± SEM. SEM, standard error of the mean. *P<0.05, **P<0.01, ***P<0.001. n.s., no significant difference.
